# Plasma metabolomics, lipidomics and cytokinomics profiling predict disease recurrence in metastatic colorectal cancer patients undergoing liver resection

**DOI:** 10.3389/fonc.2022.1110104

**Published:** 2023-01-11

**Authors:** Susan Costantini, Elena Di Gennaro, Francesca Capone, Alfonso De Stefano, Guglielmo Nasti, Carlo Vitagliano, Sergio Venanzio Setola, Fabiana Tatangelo, Paolo Delrio, Francesco Izzo, Antonio Avallone, Alfredo Budillon

**Affiliations:** ^1^ Experimental Pharmacology Unit, Istituto Nazionale Tumori - IRCCS - Fondazione G. Pascale, Napoli, Italy; ^2^ Experimental Clinical Abdominal Oncology Unit, Istituto Nazionale Tumori - IRCCS - Fondazione G. Pascale, Napoli, Italy; ^3^ Innovative Therapy for Abdominal Metastases Unit, Istituto Nazionale Tumori - IRCCS - Fondazione G. Pascale, Napoli, Italy; ^4^ Radiology Unit, Istituto Nazionale Tumori - IRCCS - Fondazione G. Pascale, Napoli, Italy; ^5^ Pathology Unit, Istituto Nazionale Tumori - IRCCS - Fondazione G. Pascale, Napoli, Italy; ^6^ Colorectal Oncological Surgery Unit, Istituto Nazionale Tumori - IRCCS - Fondazione G. Pascale, Napoli, Italy; ^7^ Hepatobiliary Surgery Unit, Istituto Nazionale Tumori - IRCCS - Fondazione G. Pascale, Napoli, Italy

**Keywords:** colorectal cancer, liver metastases, metabolomics, cytokines, NMR spectroscopy

## Abstract

**Purpose:**

In metastatic colorectal cancer (mCRC) patients (pts), treatment strategies integrating liver resection with induction chemotherapy offer better 5-year survival rates than chemotherapy alone. However, liver resection is a complex and costly procedure, and recurrence occurs in almost 2/3rds of pts, suggesting the need to identify those at higher risk. The aim of this work was to evaluate whether the integration of plasma metabolomics and lipidomics combined with the multiplex analysis of a large panel of plasma cytokines can be used to predict the risk of relapse and other patient outcomes after liver surgery, beyond or in combination with clinical morphovolumetric criteria.

**Experimental design:**

Peripheral blood metabolomics and lipidomics were performed by 600 MHz NMR spectroscopy on plasma from 30 unresectable mCRC pts treated with bevacizumab plus oxaliplatin-based regimens within the Obelics trial (NCT01718873) and subdivided into responder (R) and non-R (NR) according to 1-year disease-free survival (DFS): ≥ 1-year (R, n = 12) and < 1-year (NR, n = 18). A large panel of cytokines, chemokines, and growth factors was evaluated on the same plasma using Luminex xMAP-based multiplex bead-based immunoassay technology. A multiple biomarkers model was built using a support vector machine (SVM) classifier.

**Results:**

Sparse partial least squares discriminant analysis (sPLS-DA) and loading plots obtained by analyzing metabolomics profiles of samples collected at the time of response evaluation when resectability was established showed significantly different levels of metabolites between the two groups. Two metabolites, 3-hydroxybutyrate and histidine, significantly predicted DFS and overall survival. Lipidomics analysis confirmed clear differences between the R and NR pts, indicating a statistically significant increase in lipids (cholesterol, triglycerides and phospholipids) in NR pts, reflecting a nonspecific inflammatory response. Indeed, a significant increase in proinflammatory cytokines was demonstrated in NR pts plasma. Finally, a multiple biomarkers model based on the combination of presurgery plasma levels of 3-hydroxybutyrate, cholesterol, phospholipids, triglycerides and IL-6 was able to correctly classify patients by their DFS with good accuracy.

**Conclusion:**

Overall, this exploratory study suggests the potential of these combined biomarker approaches to predict outcomes in mCRC patients who are candidates for liver metastasis resection after induction treatment for defining personalized management and treatment strategies.

## Introduction

Colorectal cancer (CRC) is the third most common cancer worldwide and the second leading cause of cancer deaths in the western world (1, 2), with liver metastases developing in almost half of the cases with metastatic disease. Surgery for CRC liver metastases (CRCLM) is the only curative treatment, resulting in 50% 5-year survival rates when integrated with effective systemic therapies (3, 4). However, liver resection is a complex and costly procedure associated with significant morbidity and mortality risks, and relapse occurs in almost two-thirds of patients after potentially curative resection, within 2 years of the surgery in the majority of cases (5). Therefore, accurate identification of patients at higher risk of recurrence is critical for developing different follow-up schedules or avoiding nonbeneficial invasive surgical procedures.

Currently, resectability is established using clinical-morphovolumetric criteria based on conventional computed tomography (CT) or magnetic resonance imaging (MRI), approaches that cannot recognize occult metastatic disease elsewhere, thus affecting the patient outcome (6). Moreover, recent evidence and a meta-analysis do not support the routine use of preoperative positron emission tomography (PET)-CT in patients with potentially resectable disease (6, 7). Efforts to develop risk scores that include clinical parameters resulted in several proposed prognostic scoring systems that failed to be adequately predictive and are unlikely to enter clinical practice (8–10). Similarly, a few attempts have been made to study the prognostic role of tumor molecular parameters, such as mutational status or tumor gene expression profiles, with no consensus yet (9, 11, 12). Circulating blood biomarkers for prognostication are currently attracting increasing attention because they are minimally invasive and their trend can be evaluated over time.

The application of metabolomic profiling to biological fluids has recently emerged as a powerful and reliable tool for identifying novel biomarkers to improve early diagnosis and prognostication and for predicting the response of cancer patients to treatment (13–16). In this context, nuclear magnetic resonance (NMR) spectroscopy represents the only nondestructive technique able to rapidly identify and quantify complex mixtures of metabolites in small samples, and its use is increasing for successful patient stratification in various diseases, including cancer (17–20).

The NMR approach has already been used to study metabolic alterations in CRC using a variety of sample types, including urine, tissues, sera and feces (21–24). Serum metabolomics has been demonstrated to have a potential role in CRC clinical management for early detection of CRC (25–29), enhancing staging accuracy (30, 31), distinguishing locoregional disease vs. metastatic disease, differentiating between liver-only vs. extrahepatic metastases, and identifying patients who will have a poor outcome (19, 27, 32, 33).

Cytokines contribute to cancer development and progression, and deregulated serum levels of cytokines can be detected in cancer patients, including colorectal cancer, and they correlate with patient outcomes (27, 34–37).

We recently completed a phase 3 study (Obelics trial) of 230 mCRC patients, investigating different schedules of bevacizumab plus oxaliplatin/fluoropyrimidine regimens (mFOLFOX-6/mOXXEL) (38). In detail, we compared the traditional concomitant administration of bevacizumab with an experimental schedule in which bevacizumab was given 4 days before chemotherapy. Although the objective response rate, the primary endpoint of the study, did not significantly differ between the two treatment groups, a longer overall survival (OS), fewer adverse effects and better health-related quality of life were observed with the sequential bevacizumab administration schedule. A total of 81 patients enrolled in the trial underwent resection of metastases with no significant differences observed in the radical resection rate between the two arms.

Here, we retrospectively evaluated the peripheral blood samples of mCRC patients who underwent liver metastasis resection within the Obelics trial, hypothesizing that the integration of plasma metabolomics and lipidomics as well as the multiplex analysis of a large panel of plasma cytokines may enable a more informative prediction, either at diagnosis or over time, of the risk of relapse and outcome after liver surgery, beyond or in combination with clinical morpho-volumetric criteria.

## Materials and methods

### Study population and sample collection

**Figure 1:**
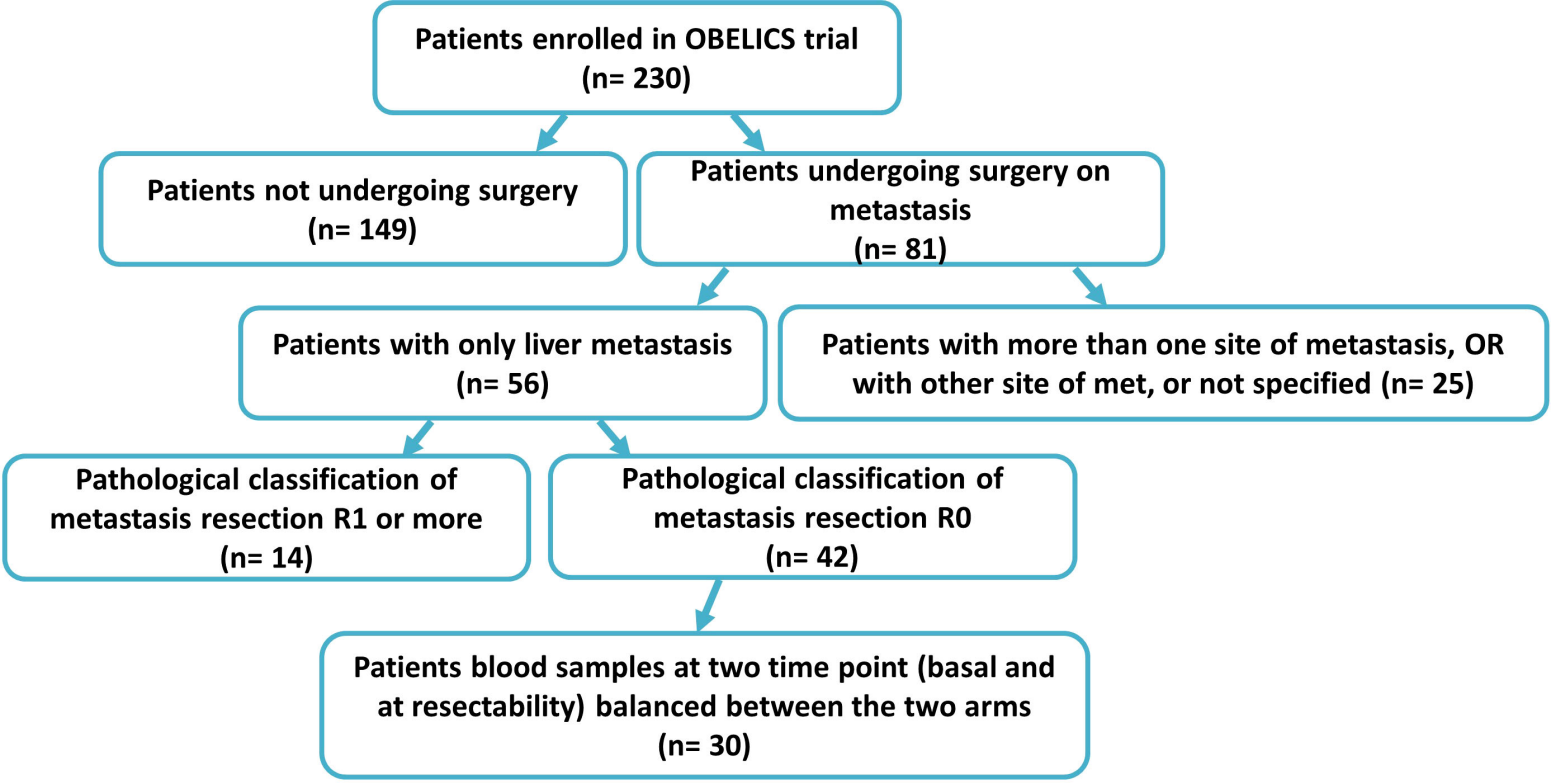
CONSORT diagram showing the 30 mCRC patients selected.

The clinical samples were collected within the multicentricObelics trial (NCT01718873), which investigated different schedules of bevacizumab in combination with oxaliplatin plus fluoropyrimidines (FOLFOX-6 or OXXEL) regimens for treating metastatic colorectal cancer (mCRC) patients. Patient recruitment and sample collection were approved by the ethics committee of the National Cancer Institute of Naples – Fondazione G. Pascale. Written informed consent was obtained from all of the patients in accordance with the Declaration of Helsinki for the use of human biological samples for research purposes. Blood samples from the patients were obtained after overnight fasting. Plasma samples were retrospectively selected from patients in the Obelics trial enrolled at Pascale Institute who underwent surgery for resection of only liver metastases; patients with severe surgical complications were excluded. For thirty patients (15 in the experimental arm and 15 in the standard arm) with these characteristics, blood samples were collected at different time points (baseline and whenresectability was defined at the response evaluation). The patient characteristics are shown in **Table 1**, and the patient inclusion process is reported in **Figure 1**. The patients were subdivided into responder (R) and non-R (NR) according to 1-year disease-free survival (DFS): ≥ 1-year (R, n = 12) and < 1-year (NR, n = 18).

**Table 1:** Baseline characteristics of 30 patients.

	Patients (#30)	Good outcome – R (#12)	Pour outcome –NR (#18)	p-value
Gender				
M	12(40%)	2 (16.7%)	10 (55.6%)	0.0332*
F	18 (60%)	10 (83.3%)	8 (44.4%)	
**Age median** **(95% CI range)**	59 (54.9-61.6)	55 (52.1-60.2)	61 (54.5-64.8)	
**PFS median** **(95% CI range)**	10.43 (11.69-27.72)	47.39 (31.62-52.65)	3.015 (2.75-6.76)	
RAS status				
wild-type	11 (36.7%)	3 (25%)	8 (44.4%)	0.279
mutated	19 (63.3%)	9 (75%)	10 (55.6)	
TRG				
1-2	19 (63.3%)	10 (83.3%)	9 (50%)	0.0634
3-4	11 (36.7%)	2 (16.7%)	9 (50%)	
ARM				
esperimental	15 (50%)	5 (41.7%)	10 (55.6%)	0.456
standard	15 (50%)	7 (58.3%)	8 (44.4%)	
CEA				
> 5UI/L	23 (70%)	10 (80%)	13 (72%)	0.480
≤ 5 UI/L	7 (30%)	2 (20%)	5 (18%)	
Primary tumor location				
right colon	10 (33.3%)	5 (41.7%)	5 (27.8%)	0.429
left colon	20 (66.7%)	7 (58.3%)	13 (72.2%)	

Significant p-value < 0.05 is indicated by symbol *.

Blood samples were collected in plasma preparation Vacutainer tubes (BD Biosciences), centrifuged at 1500 × g for 10 min within 2 h of collection and then stored at -80°C until the day of analysis.

Vital tumor areas selected by a pathologist from the metastases resected from the liver of ten patients were frozen at -80°C until analysis. Normal liver tissues for the same patients were also collected and frozen.

Pathological tumor response was evaluated according to the 2010 American Joint Committee on Cancer (AJCC) TRG system: TRG 1, no viable cancer cells; TRG 2, single or few cancer cells; TRG 3, fibrosis predominating over residual cancer; TRG 4, predominant viable cancer cells outgrowing the fibrosis (39).

### Plasma ^1^H NMR spectroscopy

All plasma samples were prepared for NMR analysis by mixing 330 μL of plasma with 300 μL of PBS (containing 10% v/v H_2_O) and 70 μL of reference standard D_2_O solution containing 0.1 mM sodium 3-trimethylsilyl [2,2,3,3-2H4] propionate (TSP). Samples were inserted into an NMR tube, and all of the spectra were recorded using a Bruker Avance III HD (600 MHz) NMR spectrometer operated at a 599.97 MHz ^1^H resonance frequency and equipped with a TCI cryoprobe. To attenuate the broad NMR signals from the slowly tumbling molecules in the lipids and proteins, a standard Carr−Purcell−Meiboom−Gill (CPMG) pulse sequence was used to record the ^1^D spin−echo spectra. To suppress the water peaks, a CPMG presaturation pulse sequence was used with the equation *-RD-90°-(t-180°-t) n - ACQ*, where *RD* is the relaxation delay of 2 s; 90° and 180° represent the pulses that trip the magnetization vector; *t* is the spin−echo delay; *n* represents the number of loops; and *ACQ* is the data acquisition period. In our experiment, the data points were acquired using 256 transients.

### Extraction of the lipidic fractions from the plasma samples and ^1^H NMR spectroscopy

Each 100 µL plasma sample was resuspended in 170 µL of H_2_O and 700 µL of methanol. Then, 350 µL of chloroform was added, and the samples were mixed on an orbital shaker on ice for 10 min. Then, 350 μL of H_2_O/chloroform (1:1, v/v) was added to each sample and centrifuged at 4000 rpm for 10 min at 4°C. Thereafter, the lipidic (apolar) phases were collected and evaporated. Then, these fractions were dissolved in 700 µL of deuterated chloroform containing 0.1 mM TSP and inserted into NMR tubes. ^1^H-NMR spectra at 300 K were acquired using a Bruker Avance III HD (600 MHz) NMR spectrometer equipped with a TCI cryoprobe and zgesgp as the pulse sequence. The data points were acquired using 512 transients.

### Tissue ^1^H HRMAS NMR spectroscopy

Frozen tumor tissue samples were cut to an appropriate size (mean weight: 10 mg) and placed in 50 μL disposable rotor insets filled with reference standard D_2_O solution containing 0.1 mM TSP for the field lock. Inserts with frozen samples were transferred to 4 mm zirconium rotors. Samples were kept at 277 K to slow down tissue degradation. Spectra were acquired by a Bruker Avance III HD (600 MHz) NMR spectrometer equipped with a high resolution magic angle spinning (HRMAS) probe using a magic angle spinning rate of 4 kHz and CPMG presaturation pulse sequence. A total of 256 scans were collected.

### NMR data processing

All of the ^1^H NMR spectra were manually phased and baseline-corrected and referenced to the CH_3_ resonance of TSP at 0 ppm. The spectral 0.50-8.60 ppm region of the ^1^H-NMR spectra was integrated in buckets of 0.04 ppm by the AMIX package (Bruker, Biospin, Germany). In detail, we excluded, in the case of the polar spectra, the water resonance region (4.5-5.2 ppm) during the analysis and normalized the bucketed region to the total spectrum area using Pareto scaling by the MetaboAnalyst v5.0 tool (40).

### Cytokinome evaluation

A large panel of cytokines, chemokines, and growth factors were evaluated in plasma collected when resectability was defined using LuminexxMAP-based multiplex bead-based immunoassay technology. In detail, the concentrations of β-NGF, CCL2 (MCP-1), CCL3 (MIP-1α), CCL4 (MIP-1α), CCL7 (MCP-3), CCL11 (Eotaxin), CTACK (CCL27), CXCL1 (GRO-α), CXCL9 (MIG), CXCL10 (IP-10), CXCL12 (SDF-1α), FGFbasic, G-CSF, GM-CSF, HGF, IFN-α2, IFN-γ, IL-1α, IL-1ß, IL-1ra, IL-2, IL-2Rα, IL-3, IL-4, IL-5, IL-6, IL-7, IL-8, IL-9, IL-10, IL-12 (p40), IL-12 (p70), IL-13, IL-15, IL-16, IL-17, IL-18, LIF, M-CSF, MIF, PDGF-ßß, RANTES, SCF, SCGF-ß, TNF-α, TNF-β, TRAIL and VEGF were determined using the Bio-Plex Pro™ Human Cytokine Screening Panel, 48-Plex assay and a Bio-Plex array reader (Luminex, Austin, TX, USA) that quantifies multiplex immunoassays in a 96-well format with very small fluid volumes. The analyte levels were calculated using a standard curve with software provided by the manufacturer (Bio-Plex Manager Software).

### Pathway analysis of significant metabolites

Pathway analysis of the modulated metabolites was performed using the Metaboanalyst 5.0 tool (40). In detail, we calculated the centrality through Pathway Impact, a combination of the centrality and pathway enrichment results. Metabolites were selected by evaluating both VIP values > 1 in the class discrimination and correlation values >0.8. Moreover, the *Homo sapiens* pathway library was chosen and analyzed using Fisher’s exact test for overrepresentation and relative betweenness centrality for pathway topology analysis.

### Data processing and statistical analysis

The sparse partial least squares-discriminant analysis (sPLS-DA) algorithm was applied to explain the maximum separation between the defined class samples in the data. Score and loading plots were used to highlight and assess the role of X-variables (NMR signals and cytokine concentrations) in the classification models and, hence, to identify the top 10 significant NMR signals and cytokines. In detail, for the loading plot, we set H = K - 1, where H is the number of dimensions and k is the number of variables to select on each dimension (41). The significant NMR signals were assigned to metabolites and lipids using the reference metabolite spectra from the HMDB database (42).

The levels of proton signals were normalized to the total spectrum area using Pareto scaling with the MetaboAnalyst v5.0 tool (40). The average rate of change (Δ) values were obtained considering for each metabolite the ratio between [the average level of the proton signals at the response evaluation] and [the average level of the proton signals at baseline]×100 in the R and NR patient groups.

Receiver operating characteristic (ROC) curves were calculated for metabolites/lipids/cytokines that were found to be significantly correlated with DFS ≥ 1 year by the Biomarker Analysis tool of Metaboanalyst v5.0 (40). The area under the curve (AUC) was used to assess the accuracy. The 95% confidence intervals (CIs) were calculated to compute the optimal cutoffs for any given feature (significant metabolites, lipids and cytokines).

DFS was defined as the time from liver metastasis resection to the date of progression or death, whichever occurred first. Patients who did not progress were censored on the date of the last follow-up visit. OS was defined as the time from randomization to the date of death. Patients alive at the time of the final analysis were censored on the date of the last follow-up information available. DFS and OS curves were estimated according to the Kaplan−Meier method, and differences were evaluated with the log-rank test in MedCalc software (https://www.medcalc.org).

The Cox regression model was used to assess the role of the cutoff for metabolite parameters in predicting DFS and OS. Hazard ratios (HR) were derived from the Cox regression analysis, and their 95% confidence intervals (95% CI) were calculated using the proportional hazard model. Univariate analysis assessed the correlation of the baseline patient characteristics (sex, CEA, RAS status, TRG, treatment ARM and primary tumor location), metabolites, lipids and cytokines with DFS and OS. In all statistical tests, a p value less than 0.05 was considered significant. A multivariate analysis was performed using MedCalc software (https://www.medcalc.org) according to a backward elimination of factors showing a p value less than 0.05 in the univariate analysis.

Finally, biomarker analyses were performed on the basis of ROC curves for multiple biomarkers (metabolites, lipids and cytokines) using the support vector machine (SVM) algorithm by the module “Biomarker Analysis” in the Metaboanalyst 5.0 tool (40). The SVM classification algorithm aims to find a nonlinear decision function in the input space by mapping the data into a higher dimensional feature space and separating it by means of a maximum margin hyperplane (43).The input of an SVM is a training set S= (x_1_, y_1_)…,(x_n_, y_n_) of the vector of features (metabolites, lipids and cytokines) for each pt together with their known classes y_i_∈ {R, NR}. The output of an SVM is a Model f: X → {R, NR} that predicts the class f(x) of any new pt (44). MetaboAnalyst’s SVM analysis is performed through recursive feature selection and sample classification using a linear kernel (45). Features are selected based on their relative contribution to the classification using cross validation error rates. The least important features are eliminated in the subsequent steps. This process creates a series of SVM models. The features used by the best model are considered to be important and are ranked by their frequencies of being selected in the model. In detail, in our study, different biomarker models were tested, and sample predictions were made. We evaluated 100 cross validations (CVs) to produce a smooth ROC curve, and the results were averaged to generate the plot. The average of the predicted class probabilities of each sample across the 100 cross-validations was produced (40).

## Results

### Metabolic profiles of plasma samples from metastasis-resected cancer patients

Blood samples from a group of mCRC patients undergoing liver metastasis resection after first-line conversion oxaliplatin-based chemotherapy plus bevacizumab, enrolled within the Obelics trial (NCT01718873), were collected at baseline and at the time of response evaluation when resectability was established. Only those patients obtaining R0 resection without any severe surgical complications were considered, balanced between the two arms (**Figure 1**). Blood samples available from thirty patients were analyzed by comparing, on the basis of disease-free survival (DFS) at 1 year: good responders (R), with a DFS ≥ 1 year, versus poor responders (NR), with a DFS < 1 year. The median DFS was 47.39 months (95% CI, 31.62-52.65) and 3 months (95% CI, 2.75-6.76) for R (n=12) and NR (n=18) patients, respectively. Of note, the median follow-up in this patient population was 39 months.

Baseline patient and tumor characteristics were well balanced between the two groups, although there was a statistically significant difference in the gender proportion (**Table 1**). The median number of chemotherapy cycles administered before surgery was 6 (range 6-12) in both groups of patients; only 22% of NR and 25% of R patients received 12 cycles.

**Figure 2:**
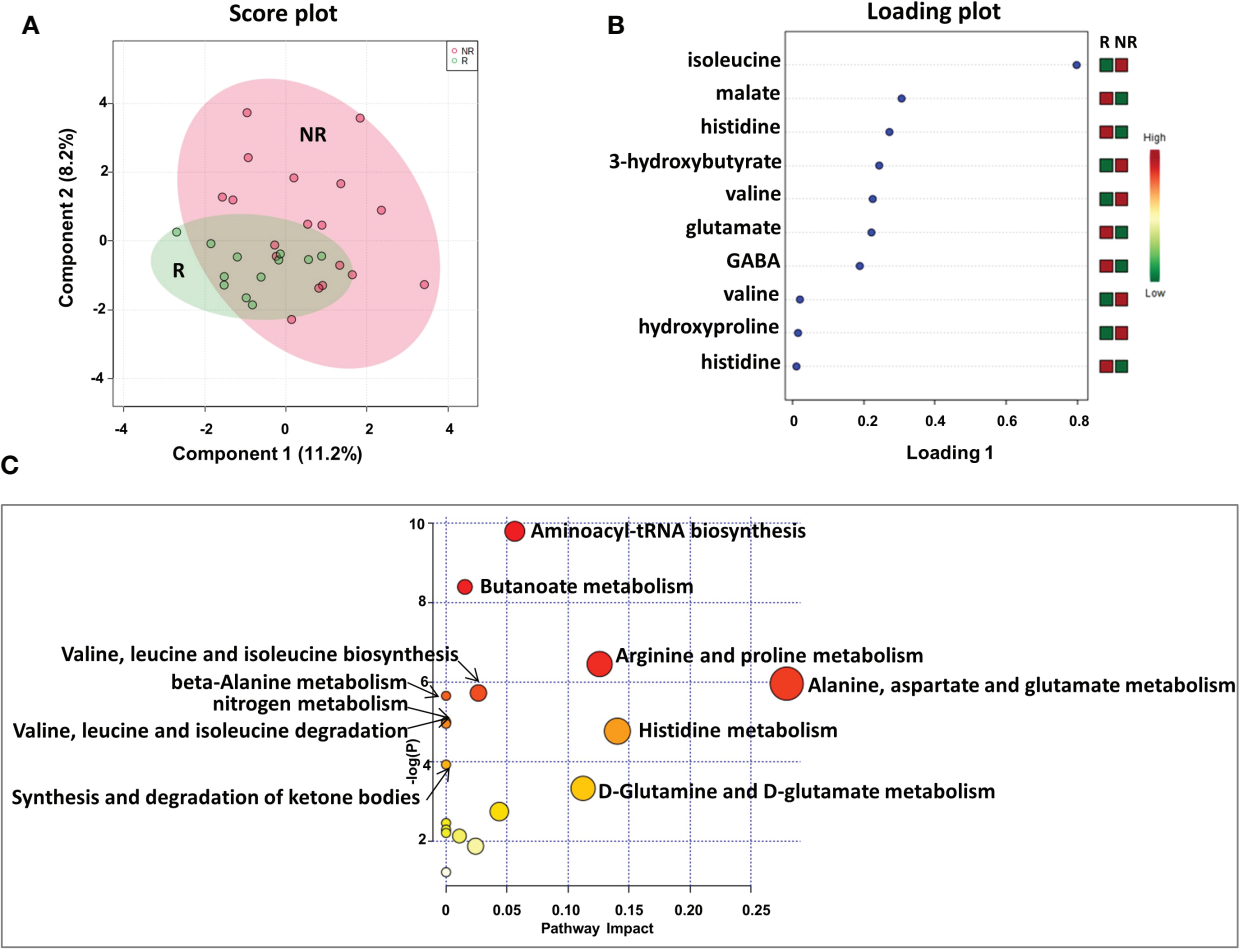
Score plot **(A)** and loading plot **(B)** related to metabolomic profiling on plasma of mCRC patients, collected at response evaluation when liver resectability was established and subdivided accordingly to DFS in good (R; DFS ≥ 1 year) and bad (NR; DFS < 1 year) responders. **(C)** The most significant pathways are reported: colors, from yellow to red, indicate increasing levels of statistically significance (p values from the pathway enrichment analysis); size of the nodes indicates pathway impact (a combination of both pathway enrichment results and centrality of each of the matched metabolites within the pathway).

We first analyzed the plasma metabolic signature at the time of response evaluation when resectability was established. As reported in **Figure 2**, sparse partial least squares discrimination analysis (sPLS-DA) (19.4% of the total variance), calculated on the ^1^H NMR plasma spectra, clearly discriminated R from NR patients (**Figure 2A**), with a model accuracy of 63.3%, suggesting that the two study groups are distinctively different in terms of their plasma metabolic profiles. Moreover, the sPLS-DA score plot showed that the profiles of the R patients clustered together, whereas the NR patients were scattered, suggesting that patients with good outcomes may have similar metabolic profiles (**Figure 2A**).

An analysis of the PLS loading was then conducted to identify the metabolites found to be most relevant to the class separation (as reported in the Methods section). As shown in the loading plot of the top 10 NMR signals that were significantly different, it appears that R patients were characterized by lower plasma levels of isoleucine, 3-hydroxybutyrate, valine and hydroxyproline and higher levels of malate, histidine glutamate and gamma-aminobutyric acid (GABA) (**Figure 2B**).Notably, two NMR signals for both valine and histidine were reported, reinforcing the significance of their differential expression between the two patient groups.

Furthermore, these metabolites were used to perform a metabolite-set enrichment analysis. A complex interplay of several different metabolic pathways and metabolites was highlighted (**Figure 2C**; **Table 1S**). In detail, aminoacyl-tRNA biosynthesis; butanoate metabolism; arginine and proline metabolism; alanine, aspartate and glutamate metabolism; valine, leucine and isoleucine biosynthesis; beta-alanine metabolism; nitrogen metabolism; valine, leucine and isoleucine degradation; histidine metabolism; synthesis and degradation of ketone bodies; and D-glutamine and D-glutamate metabolism emerged as playing a role in discriminating the plasma metabolic profiles of R from NR patients.

Next, to establish the optimal cutoff value for the metabolites selected by sPLS-DA, we performed ROC curve analysis, finding areas under the curve (AUC) values of the metabolites ranging between 0.63 and 0.74 (**Figure 1S**). Based on the metabolite parameter cutoff values, univariate and multivariate analyses were then conducted to evaluate metabolites potentially associated with DFS.

**Figure 3:**
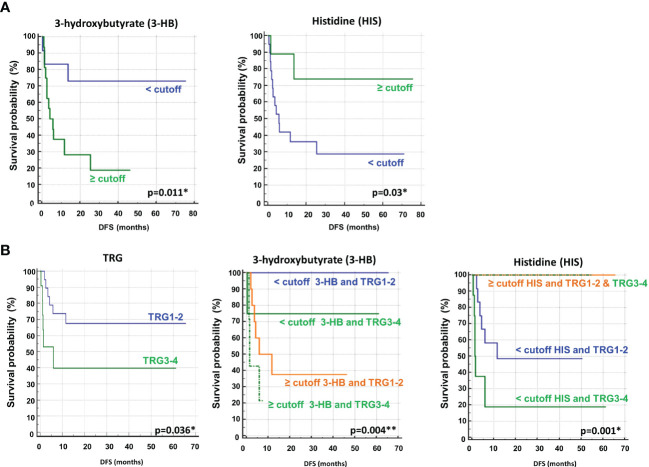
**(A)** Kaplan–Meier curves of disease free survival (DFS) accordingly to 3-hydroxybutyrate (3-HB) and histidine (HIS) **(B)** Kaplan–Meier curves of DFS accordingly to tumor regression grade (TRG) alone or in combination with either 3-HB or HIS. Log-rank p-values are reported. * and ** symbols indicate p-values < 0.05 and < 0.01, respectively.

Univariate analysis demonstrated that sex (M vs. F) (HR, 2.90; 95% CI, 0.94–8.91; *P*=0.028) and tumor regression grade (TRG, 3-4 vs. 1-2) (HR, 2.80; 95% CI, 0.85–9.21; *P*=0.036) were significantly associated with DFS (**Table 2**). No significant association was found between DFS and RAS status, CEA, primary tumor location, or treatment arm. Among the metabolites, both 3-hydroxybutyrate (HR, 4.35; 95% CI, 1.58–11.97; *P*=0.011) and histidine (HR, 0.23; 95% CI, 0.081–0-63; *P*=0.03) predicted DFS (**Table 2**, **Figure 3A**). In detail, as shown by the Kaplan−Meier survival curves, only lower levels of 3-hydroxybutyrate (<cutoff) or higher levels of histidine (≥cutoff), evaluated before surgery, correlated with a more favorable DFS (**Figure 3A** and **Figure 2S**). These two metabolites were also the only parameters significantly correlated with overall survival (OS) in the univariate analysis (**Table 2S**, **Figure 3S**). Notably, in multivariate analysis, 3-hydroxybutyrate was the only parameter that significantly predicted DFS (**Table 2**) and OS (**Table 2S**).

**Table 2:** Univariate and Multivariate analyses of baseline patients characteristics, metabolites, lipids and cytokines for disease free survival (DFS).

	Univariate	Multivariate
	HR (95% CI) P value	HR (95% CI) P value
Patients characteristics		
Gender		
(M *vs* F)	2.90 (0.94-8.91) p=0.028*	1.21 (0.78-1.86)p=0.30
RAS status		
(mut vs wt)	1.42 (0.49-4.41) p=0.54	–
TRG		
(3-4 *vs* 1-2)	2.80 (0.85-9.21) p=0.036*	1.65 (1.04-2.61)p=0.09
ARM		
(standard *vs* experimental)	2.37 (0.76-7.37) p=0.36	–
CEA		
(>5 UI/L vs ≤ 5 UI/L)	1.16 (0.30-4.53) p=0.83	–
Primary tumor location		
(left *vs* right)	1.01 (0.29-3.44) p=0.86	–
Metabolites(nps)		
3-hydroxybutyrate level		
(≥-0.322 vs < -0.322)	4.35 (1.58-11.97) p=0.011*	8.34 (1.00-69.34) p=0.020*
histidine level		
(<0.158 vs ≥0.158)	4.42 (1.58-12.39)p=0.03*	1.95 (0.84-16.7) p=0.96
Lipids(nps)		
Cholesterol		
(≥0.0109 *vs <*0.0109)	9.72 (3.47-27.20) p=0.005*	1.26 (0.27-3.54) p=0.67
Triglycerides		
(≥-0.000524 vs <-0.000524)	4.51 (1.59-12.81) p=0.003*	1.5 (0.33-9.67) p=0.56
Phospholipids		
(≥- 0.147 vs <- 0.147)	2.83 (1.01-7.90) p=0.042*	1.43 (0.061-33.65) p=0.79
Cytokines(pg/mL)		
IL-6		
(≥5.45 *vs <*5.45)	4.83 (1.68-13.83) p=0.002*	1.11 (0.18-3.73) p=0.55
SCGF-β		
(≥80000 *vs *<80000)	6.44 (1.83-22.63) p=0.034*	1.89 (0.41-19.25) p=0.59
CXCL10		
(≥189 *vs <*189)	7.98(2.43-26.20) p=0.014*	1.05 (0.96-3.64) p=0.95
CTACK		
(<6.30 *vs* ≥6.30)	6.26 (0.61-63.72) p=0.022*	1.65 (0.79-2.54) p=0.11

HR, hazard ratio; CI, confidence interval; M, male; F, female; nps, normalized values of the proton signals. Significant p-values <0.05 are reported in bold.

**Figure 4:**
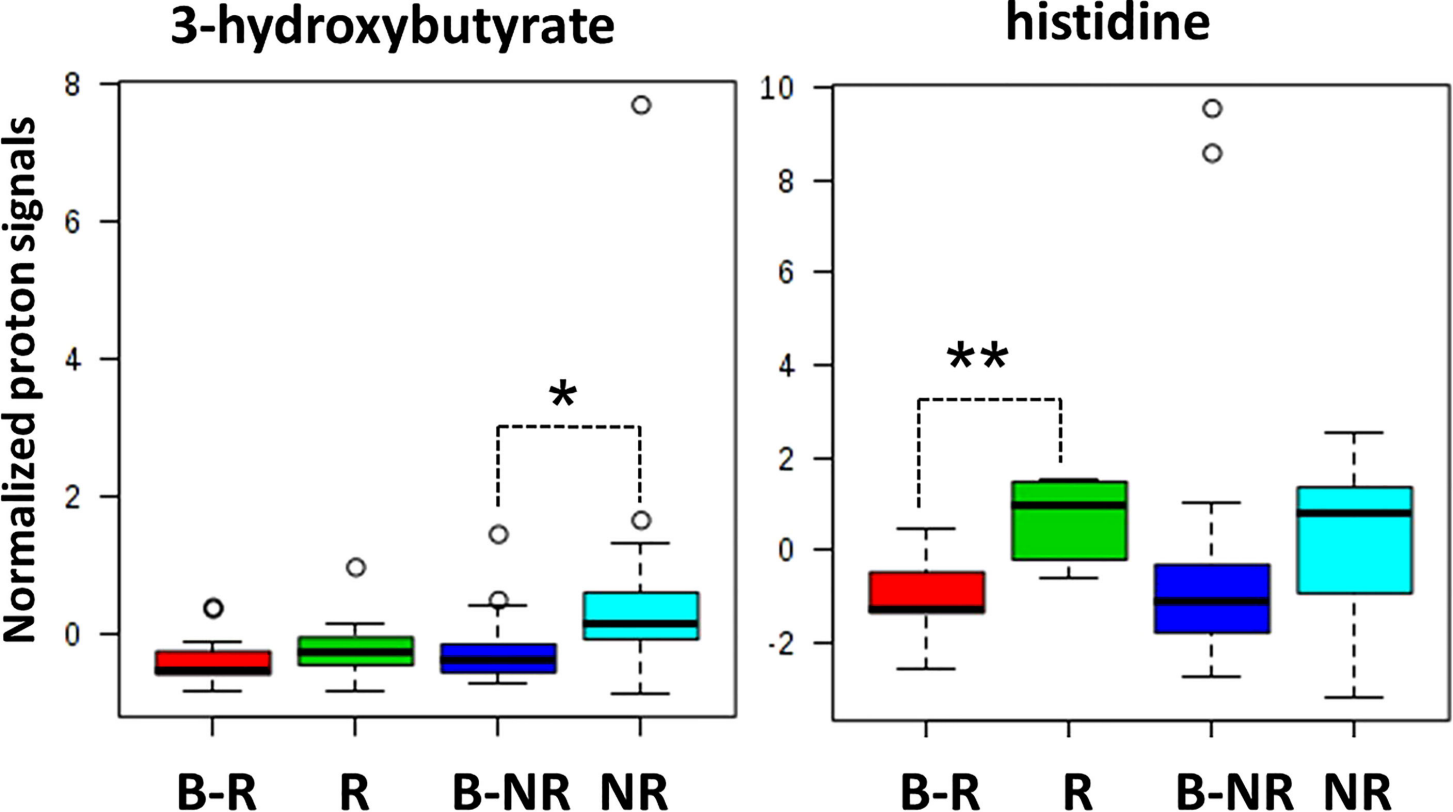
Box and whisker plots summarize the normalized values of 3-hydroxybutyrate and histidine evaluated at both baseline (B-R and B-NR) and at response evaluation (R and NR) (* p-value=0.042; ** p-value=0.001).

The prognostic role of TRG in patients with locally advanced rectal cancer treated with neoadjuvant chemoradiation has been explored and was previously confirmed as a predictor of disease-free survival in this setting (46, 47). Recently, TRG has been suggested as a useful prognostic factor in mCRC patients subjected to preoperative chemotherapy before metastasis resection (48). This observation was confirmed in our cohort of patients, where poor pathological responses (TRG 3-4) were associated with shorter DFS than complete and near-complete responses (TRG 1-2) (**Table 2**, **Figure 3B**). When the metabolites histidine or 3-hydroxybutyrate were combined with TRG status, a striking separation of distinct categories was obtained (**Figure 3B**). Indeed, the two metabolites were far better predictors of DFS, with patients with either low 3-hydroxybutyrate or high histidine being associated with more favorable DFS outcomes, independent of TRG status (**Figure 3B**). Conversely, the patients with high 3-hydroxybutyrate or low histidine levels, although they had favorable prognostic TRG1-2, displayed a worse prognosis, further highlighting the powerful role of both metabolites in predicting DFS (**Figure 3B**). Similar data were also obtained for OS (**Figure 4S**). Kaplan–Meier curves of DFS and OS related to TRG in combination with other metabolites confirmed that only low 3-hydroxybutyrate or high histidine levels were associated with more favorable DFS/OS outcomes, independent of TRG status. The curves obtained for isoleucine were reported as representative example (**Figure S5**).

When we considered the plasma metabolic signature at baseline, the sPLS-DA score plot demonstrated a less evident discrimination between R and NR patients (**Figure 6SA**). However, among the top 10 NMR signals contributing to class separation, the PLS loading plot again identified high levels of 3-hydroxybutyrate, hydroxyproline, and isoleucine as associated with NR patients (**Figure 6SB**), as also reported at the response evaluation time point for this group of patients (**Figure 2B**). In addition, high levels of 2-hydroxybutyrate, proline, trimethylamine and aspartate, and low levels of phosphoethanolamine and betaine, were among the most significant metabolites associated with NR patients (**Figure 6SB**). Overall, only a limited number of metabolic pathways, all included in the analysis reported in **Figure 2C**, were highlighted, confirming that the metabolic profiles of NR and R patients, at baseline, were less discriminated than those evaluated at the response evaluation time point (**Figure 6SC** and **Table 3S**).

Indeed, when we considered the normalized values of 3-hydroxybutyrate and histidine NMR signals at both baseline (B-R and B-NR) and at the response evaluation (R and NR), a clear rate of change in metabolic abundance over time from baseline was observed (**Figure 4**). Notably, the average rate of change (Δ) for 3-hydroxybutyrate levels increased by 23.9% in NR and only 3.8% in R patients from baseline (*p*=0.042); conversely, the average Δ for histidine levels increased significantly by 71.8% in R vs. only 12.4% in NR patients from baseline (*p*=0.001) (**Figure 4**). In other words, 3-hydroxybutyrate, whose high levels predicted a poor DFS at the response evaluation, increased during treatment only in the case of NR patients, whereas histidine, whose high levels at the response evaluation predicted a more favorable DFS, increased during treatment only in R patients. As reported above, in order to validate that only the 3-hydroxybutyrate and histidine were modulated in statistically significant way during treatment in NR or R patients, the plots obtained for isoleucine were reported as representative example (**Figure 7S**).

### Metabolomic profiles of cancer tissues from resected liver metastases

**Figure 5:**
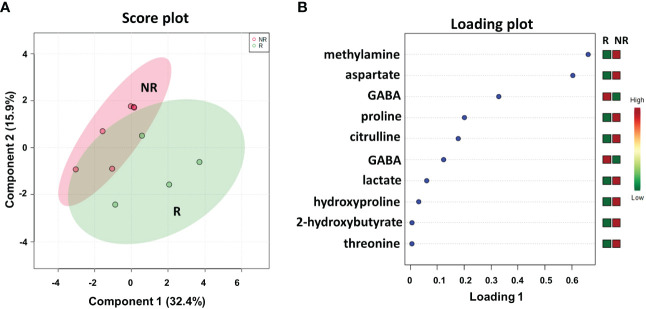
Score plot **(A)** and loading plot **(B)** related to metabolomic profiling, on resected liver metastases tissues 4 R and 6 NR mCRC subdivided accordingly to DFS in good (R; DFS ≥ 1 year) and bad (NR; DFS < 1 year) responders.

We conducted a parallel metabolomics investigation by ^1^H HRMAS NMR analysis on 10 available patient-matched frozen resected liver metastasis tissues, 6 from NR and 4 from R patients. Notably, the sPLS-DA (48.3% of the total variance) calculated on the ^1^H NMR tissue spectra clearly discriminated R from NR patients with a model accuracy of 80% (**Figure 5A**), suggesting that the two study groups are distinctively different in terms of their tissue metabolic profiles.

As shown in the loading plot (**Figure 5B**), the top 10 NMR signals were significantly different between the two patient cohorts. It is worth noting that higher levels of hydroxyproline and lower levels of GABA were observed in NR patients than in R patients, recapitulating the data obtained in the plasma samples (see **Figure 2B**). We also conducted a targeted analysis in order to verify tissue levels of 3-hydroxybutyrate and histidine metabolites that were not among the top 10 NMR signals. Interestingly, although not statistically significant, higher levels of 3-hydroxybutyrate and lower levels of histidine were confirmed in NR tissues in line with the data reported on plasma samples (**Figure 8S**).

In addition, higher levels of methylamine, aspartate, proline, citrulline, lactate, 2-hydroxybutyrate, and threonine were observed in NR patients than in R patients.

We also compared the metabolic profiles of all liver metastases (LM) with the matched adjacent noncancerous tissues (NC), again demonstrating a clear separation into two distinct clusters (**Figure 9SA**), with high levels of proline, 2-hydroxybutyrate, aspartate and lactate associated with LM vs. NC (**Figure 9SB**), which interestingly were previously reported among the top metabolites discriminating NR vs. R in LM tissues (**Figure 5**). Indeed, the metabolite-set enrichment analyses demonstrated three common pathways (aminoacyl-tRNA biosynthesis; alanine, aspartate and glutamate metabolism; valine, leucine and isoleucine biosynthesis) distinguishing both R vs. NR LM tissues and LM vs. NC tissues (**Figure 10SA**). Notably, some altered common pathways and metabolites (aminoacyl-tRNA biosynthesis; arginine and proline metabolism; alanine, aspartate and glutamate metabolism; and valine, leucine and isoleucine biosynthesis) distinguished R vs. NR patients in both plasma and metastatic tissue metabolomics, suggesting potential mechanistic correlations (**Figure 10SB**).

### Lipidomic profiles of plasma samples from metastasis-resected cancer patients

As suggested from all of the data reported above, plasma metabolic profiling at the response evaluation time point is able to discriminate R from NR patients, reflecting the impact of treatment. Therefore, further analyses conducted on plasma samples from our cohort of patients were limited to this time point.

**Figure 6:**
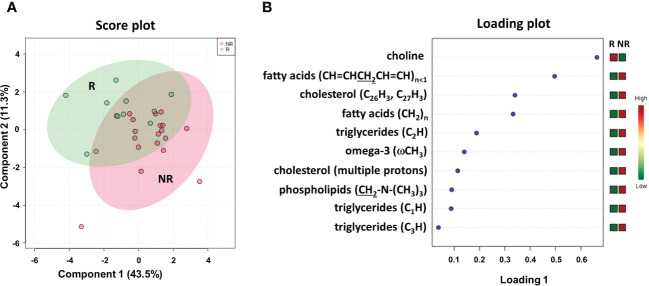
Score plot **(A)** and loading plot **(B)** related to lipidomic profiling on plasma of mCRC patients, collected at response evaluation when liver resectability was established and subdivided accordingly to DFS in good (R; DFS ≥ 1 year) and bad (NR; DFS < 1 year) responders.

To better define a metabolic signature predicting DFS, we also acquired ^1^H NMR spectra on the lipidic fractions extracted from the thirty plasma samples. The sPLS-DA plot (54.8% of the total variance) grouped R and NR patients into two different clusters with a model accuracy of 65%, suggesting the presence of some lipidic proton signals with significantly different levels between the two patient groups (**Figure 6A**). The related loading plot showed that the NR group had lower levels of choline and higher levels of proton signals of fatty acids, cholesterol, triglycerides, omega-3 and phospholipids than the R group (**Figure 6B**), indicating increased plasma lipids in patients with poor DFS.

**Figure 7:**
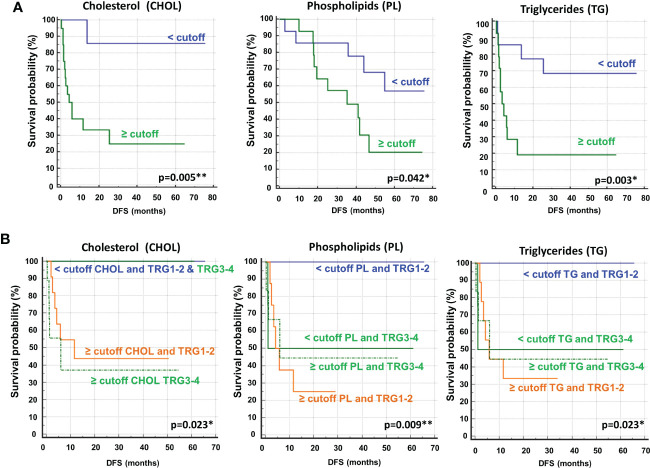
**(A)** Kaplan–Meier curves of disease free survival (DFS) accordingly to cholesterol (CHOL), phospholipids (PL) and triglycerides (TG). **(B)** Kaplan–Meier curves of DFS accordingly to tumor regression grade (TRG) alone (log-rank p=0.036) and in combination with CHOL, Pl or TG (log-rank p-values are reported). * and ** symbols indicate p-values < 0.05 and < 0.01, respectively.

To determine the optimal cutoff value for the significant lipidic signals selected by sPLS-DA, we performed ROC curve analysis (**Figure 11S**), which showed AUC values ranging between 0.681 and 0.787. Based on the obtained parameter cutoff values, univariate analysis showed that lower levels (<cutoff) of cholesterol (HR, 9.72; 95% CI, 3.47–27.20; *P*=0.005), triglycerides (HR, 4.51; 95% CI, 1.59–12.81; *P*=0.003), or phospholipids (HR, 2.83; 95% CI, 1.01–7.90; *P*=0.042) were significantly associated with good DFS (**Table 2**; **Figure 7A**) and OS (**Table 2S**; **Figure 12S**). Moreover, higher levels of choline (≥cutoff) were also found to correlate with good OS (**Table 2S**; **Figure 12S**). None of these signals were statistically significant in the multivariate analysis for both DFS and OS (**Tables 2** and **2S**).

Most importantly, as also reported for histidine and 3-hydroxybutyrate (**Figure 3**), we compared the DFS prediction potential of the lipid NMR signals with TRG, demonstrating that low cholesterol levels were a better predictor of DFS, independent of TRG status, and that both phospholipids and triglycerides plus TRG evaluation were better predictors of DFS than TRG alone (**Figure 7B**). Interestingly, the patients with high levels of either phospholipids or triglycerides, although they had favorable prognostic TRG1-2, displayed a worse prognosis, further highlighting the powerful role of lipid metabolites in DFS prediction (**Figure 7B**). Similar data for phospholipids and triglycerides were also obtained for OS (**Figure 13S**).

### Cytokinomic profiles of plasma samples from metastasis-resected cancer patients

**Figure 8:**
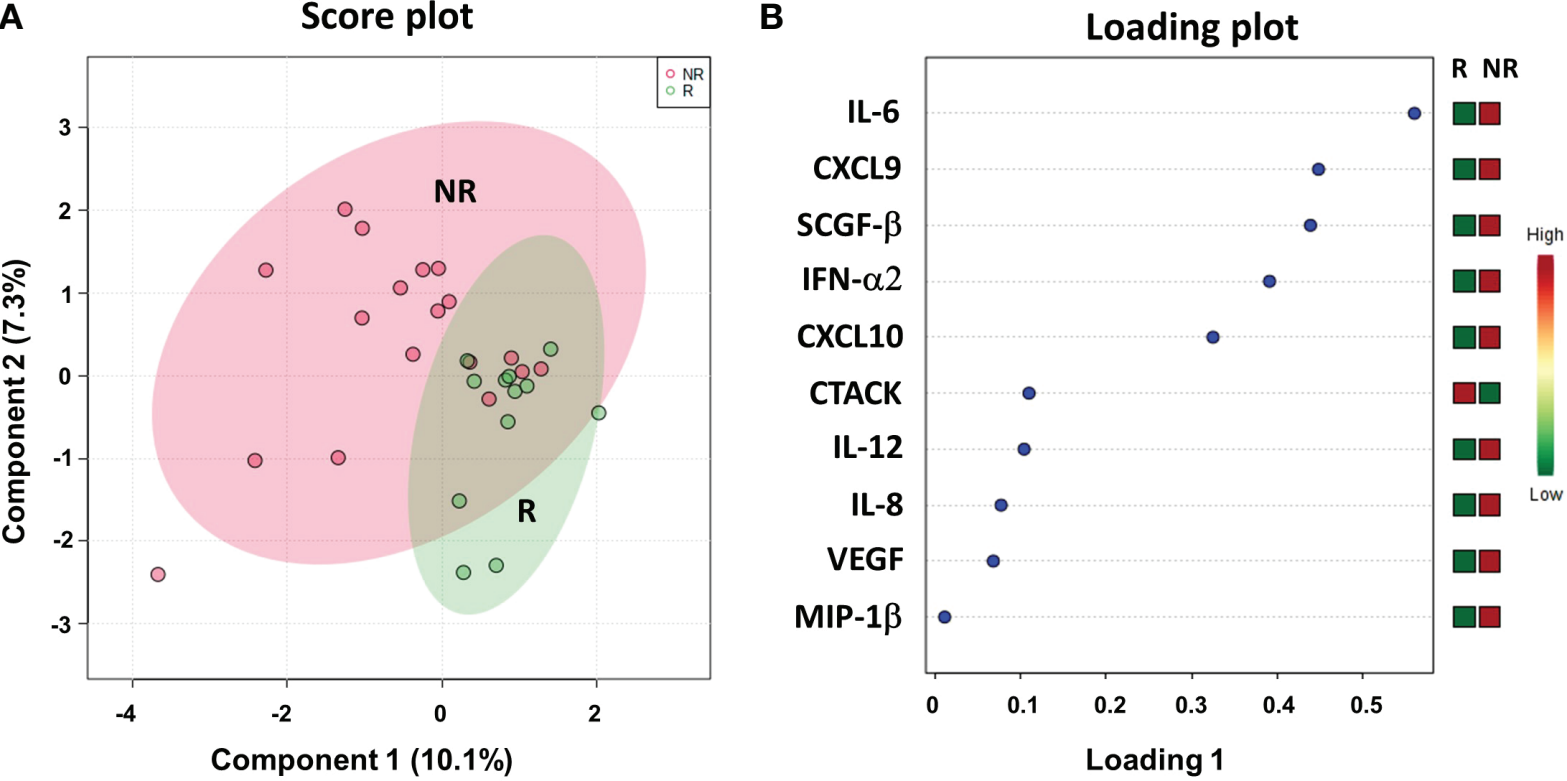
Score plot **(A)** and loading plot **(B)** related to cytokinomics profiling on of mCRC patients, collected at response evaluation when liver resectability was established and subdivided accordingly to DFS in good (R; DFS ≥ 1 year) and bad (NR; DFS < 1 year) responders.

We evaluated a panel of 48 chemokines and cytokines in the patients’ plasma at the time of the response evaluation by a multiplex bead–based system. We applied sPLS-DA to analyze the results (17.4% of the total variance), again finding that the NR and R patients grouped into two distinct clusters with a model accuracy of 66.7% (**Figure 8A**). The loading plot showed that 9 out of the top 10 cytokines that were more statistically relevant for class separation, IL-6, CXCL9 (MIG), SCGF-β, IFN-α2, CXCL10 (IP-10), IL-12, IL-8, VEGF and MIP-1β, showed higher plasma levels in the NR relative to the R patients. Only the plasma levels of CTACK chemokine (CCL27) were higher in the R group **Figure 8B**.

**Figure 9:**
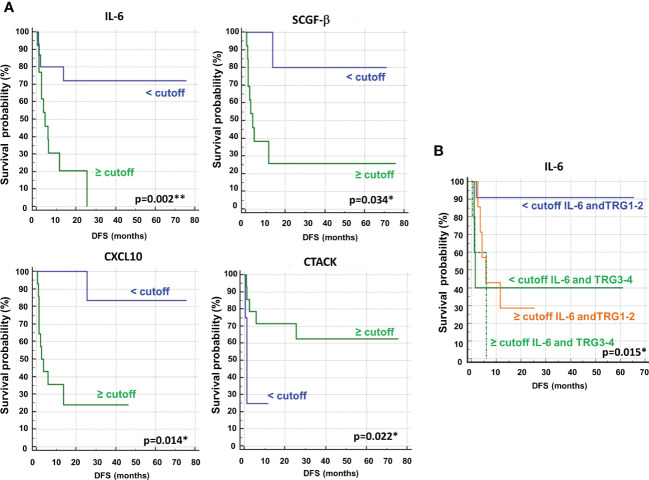
**(A)** Kaplan–Meier curves of disease free survival (DFS) accordingly to IL-6, SCGF-β, CXCL10 and CTACK. **(B)** Kaplan–Meier curves of DFS accordingly to tumor regression grade (TRG) alone (log-rank p=0.036) and in combination with IL6 (log-rank p-value is reported). * and ** symbols indicate p-values < 0.05 and < 0.01, respectively.

Next, we performed ROC curves to determine the optimal cutoff value of these cytokines, reporting AUC values ranging between 0.471 and 0.773 (**Figure 14S**). Based on the obtained parameter cutoff values, univariate analysis showed that lower levels (< cutoff) of IL-6 (HR, 4.83; 95% CI, 1.68-13.83; *P*=0.002), SCGF-β (HR, 6.44; 95% CI, 1.83-22.63; *P*=0.034), and CXCL10 (HR, 7.98; 95% CI, 2.43-26.20; *P*=0.014) or higher levels (≥cutoff) of CTACK (HR, 0.22; 95% CI, 0.026-1.91; *P* =0.022) were significantly associated with good DFS (**Table 2**; **Figure 9A**). Similar results were found for OS (**Table 2S**, **Figure 15S**).

Again, we compared the DFS prediction potential of these four cytokines with TRG, demonstrating that only low IL6 levels were a clearly better predictor of DFS, independent of TRG status (**Figure 9B**). Similar data were obtained considering OS (**Figure 16S**).

### Combined biomarker signature using the support vector machine (SVM) algorithm

**Figure 10:**
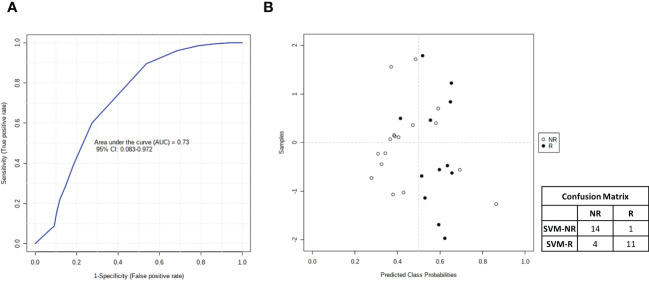
**(A)** Smooth receiving operating-characteristic (ROC) curve performed on the combination of 3-hydroxybutyrate, cholesterol, IL-6, phospholipids and triglycerides for predicting R (DFS ≥ 1 year)*vs* NR (DFS < 1 year) patients. AUC value and 95% CI are reported. **(B)** Average of predicted class probabilities of each patient group (NR and R) across the 100 cross-validations. Confusion matrix is reported in table indicated R and NR patients and those identified by support vector machines (SVM) algorithm (SVM-R and SVM-NR).

Finally, taking advantage of all of the data accumulated on metabolomics, lipidomics and cytokinomics (as predictors of DFS) associated with the DFS of our cohort of patients, we analyzed all of the possible combinations of statistically significant variables that emerged to create a multiple biomarkers model using a support vector machine (SVM) algorithm. As shown in **Figure 10**, we found that the best combination of circulating biomarkers to predict R (DFS ≥ 1 year) and NR (DFS < 1 year) patients in our cohort of metastases-resected cancer patients was represented by 3-hydroxybutyrate, cholesterol, phospholipids, triglycerides and IL-6, evaluated in plasma samples collected at the time of the response evaluation. In detail, ROC curve analysis for these combined biomarkers had an AUC value equal to 0.73 (95% CI: 0.083-0.0972) (**Figure 10A**). The combination of these five features was able to classify 15 R and 15 NR patients (**Figure 10B**) with a positive predictive value of 73% (probability of the correct identification of R) and a negative predictive value of 93% (probability of the correct identification of NR), overall correctly predicting the outcome of 83.3% (accuracy) of the patients.

## Discussion

Recurrence following chemotherapy and metastatic liver resection is a significant hurdle in CRC. Therefore, a better prediction of DFS is critical for the adequate management of patients undergoing CRCLM resection as a curative strategy. However, all previous efforts to identify risk prediction approaches beyond or in addition to clinical morpho-volumetric criteria have been quite disappointing.

Our investigation, through plasma ^1^H NMR-based metabolomics and lipidomics as well as multiplex bead-based immunoassay cytokinomics, revealed that distinct metabolites, lipids and cytokines in the plasma after conversion chemotherapy were associated with the clinical outcome in a cohort of thirty mCRC patients undergoing curative resection of liver metastases. Notably, metabolite-set enrichment analysis, evaluated in plasma at the time of response evaluation before surgery, highlighted a complex interplay between different metabolic pathways that clearly distinguished poor vs. good outcome patients.

In detail, H^1^ NMR-based plasma metabolomics profiling, evaluated at the time of response evaluation when resectability was established, identified a panel of metabolites that distinguished patients with DFS ≥ 1 and < 1 year. Moreover, according to the cutoff levels evaluated by ROC curves and the univariate analysis, two metabolites in the plasma, lower levels of 3-hydroxybutyrate and higher levels of histidine, were significantly associated with more favorable DFS and OS. Our data also demonstrated that the outcome prediction of both metabolites was better and independent from the pathological response evaluated by TRG, a recognized prognostic factor in mCRC. Notably, 3-hydroxybutyrate was the only independent factor that significantly predicted both DFS and OS in the multivariate analysis.

The plasma metabolic signature at baseline was less able to discriminate between patients with DFS ≥ 1 and < 1 year compared with the presurgery evaluation. Indeed, few metabolic pathways were able to distinguish the two groups of patients at baseline. However, the dynamic evolution of both histidine and 3-hydroxybutyrate plasma levels from baseline up to the response evaluation before surgery was consistent with their prognostic prediction. Indeed, histidine increased upon treatment significantly more in patients with DFS ≥ 1, whereas 3-hydroxybutyrate increased upon treatment only in the case of patients with DFS < 1 year.

Overall, our findings suggest that metabolomics profiling during treatment might contribute to predicting treatment resistance and tumor relapse, highlighting the importance of dynamic monitoring that offers the opportunity to modify the treatment strategy early, before surgery, which cannot be achieved with post-surgical pathology findings such as TRG.

Our results are consistent with one of the first meta-analyses analyzing serum metabolomics data in cancer patients, which found that both 3-hydroxybutyrate and histidine were among the top serum metabolites discriminating cancer patients from healthy donors across different cancer types. In detail, histidine was among the top three most decreased metabolites and 3-hydroxybutyrate was among the two most increased metabolites in cancer patient blood (49).

Histidine is an essential amino acid associated with increased inflammation and oxidative stress (50). In CRC patients, serum histidine was significantly reduced compared to healthy controls (51) and correlated with stage progression (26). Low levels of histidine have been attributed to higher activity of histidine decarboxylase, resulting in an accelerated decarboxylation of histidine to histamine, a mediator involved in inflammatory and immune responses associated with cancer initiation and progression (26).On the other hand, 3-hydroxybutyrate is a component of ketone bodies and an end-product of fatty acid β-oxidation. In this context, cancer-associated 3-hydroxybutyrate augmented levels suggest both increased protein catabolism, involving a ketogenic amino acid, and increased fatty acid oxidation, to support the energy demand of cancer cell proliferation (22).Interestingly, NMR-based metabolomics profiling studies demonstrated significantly higher serum levels of 3-hydroxybutyrate in mCRC patients versus healthy donors or CRC patients compared with those with colon polyps and healthy controls (19, 30). Notably, a recent meta-analysis of global serum metabolomics profiling studies of CRC patients compared to healthy subjects confirmed that 3-hydroxybutyrate was consistently upregulated, suggesting that, together with a few other selected metabolites, it has potential as a diagnostic biomarker for CRC (22).

Lipidomic profiling by NMR spectroscopy on the same plasma samples of mCRC patients, collected at the response evaluation, also discriminated between patients with DFS ≥ 1 and < 1 year. According to the cutoff levels evaluated by ROC curves and the univariate analysis, lower levels of cholesterol, phospholipids and triglycerides were significantly correlated with more favorable DFS and OS. In addition, higher levels of choline were correlated with OS. However, none of the lipids remained significant in multivariate analysis. Notably, as also reported for histidine and 3-hydroxybutyrate, cholesterol, phospholipids and triglycerides were independent and better predictors of DFS than TRG status.

Altered lipid metabolism is currently considered a hallmark characteristic of many cancers, including CRC (52). High levels of lipids are indeed necessary for tumor cell energy production, membrane turnover, and signal transduction, which are needed for cell growth motility and metastases (53). Elevated serum levels of cholesterol and triglycerides were previously reported in CRC patients compared to patients with benign colorectal disease and healthy controls and were correlated with advanced TNM stage (54). Both cholesterol and triglyceride serum levels were also associated with the development of distant metastasis in CRC patients (55). Notably, a recent meta-analysis including only prospective studies confirmed that high levels of total serum cholesterol and triglycerides are positively correlated with the presence of CRC (56).

Although phospholipid studies on tumors and cancer cells are limited, the concomitant downregulation of choline levels and upregulation of phospholipids associated with poor DFS in patients in our study might suggest that choline and its derivatives are consumed in greater amounts than in the normal state to drive phospholipid synthesis (57). Indeed, choline plays a critical role in the synthesis of the phospholipid components of the cell membranes, and its abnormal metabolism is emerging as being associated with oncogenesis (57, 58). Notably, in accordance with our data, lower levels of choline were found in CRC patients than in healthy donors and were correlated with stage progression (26). A very recent report showed that multiple circulating lysophosphatidylcholines (lysoPCs) and phosphatidylcholines (PCs) were associated with a high risk of disease recurrence within 6 months in patients undergoing CRCLM resection (59).

Intriguingly, altered levels of lipids in tumor cells, particularly phospholipids and cholesterol, have been suggested to promote drug resistance by altering the membrane composition (56).High lipid levels may also promote cancer development by inducing an inflammatory response and cytokine dysregulation (60). Mechanistically, crosstalk between lipid metabolism dysregulation and proinflammatory cytokine secretion has been described (61). Thus, our lipidomic findings may reflect an increased inflammatory status in patients with short DFS, in agreement with a previous report correlating an altered ^1^H-NMR lipid profile with short OS in mCRC patients (19). In this regard, some studies reported that CRC development is accompanied by cytokine production alterations (36), and a novel cytokine-based prognostic classifier has been recently developed in this setting (37).

Therefore, in our study, we also evaluated a panel of 48 chemokines and cytokines in the plasma of thirty patients at the time of the response evaluation by a multiplex bead–based system. According to the cutoff levels evaluated by ROC curves and the univariate analysis, lower levels of IL-6, SCGF-β and CXCL-10 and higher levels of CTACK correlated with a more favorable DFS and OS. In comparison to TRG, only IL-6 was an independent and better predictor of DFS. None of the cytokines evaluated remained significant after multivariate analysis.

IL-6 is a proinflammatory cytokine involved in cancer growth, invasion, progression and metastasis (62). Elevated IL6 levels in CRC patient serum (63) or tumor tissue (64) were correlated with advanced stages and a poor prognosis.

CXCL-10 is a small (10 kDa) secretable chemokine that mediates adaptive inflammation, immunity, leukocyte trafficking, and angiogenesis and induces the chemotaxis of various subtypes of leukocytes, including NK cells, T and B lymphocytes, macrophages and dendritic cells, by engaging its receptor CXCR3. CXCL-10 levels increased significantly in CRC patients compared to control subjects (65), and a recent meta-analysis revealed significant associations between low CXCL-10 expression and good overall, disease-free and relapse-free survival of CRC patients (66).

Stem cell growth factor-β (SCGF-β) is a secreted sulfated glycoprotein that functions as a growth factor for primitive hematopoietic progenitor cells. SCGF-β elevated plasma levels were associated with circulating tumor cell (CTC)-positive primary breast cancer patients, whereas interestingly, an inverse correlation with CTCs was observed for Cutaneous T-cell attracting chemokine (CTACK) in the same patient cohort (67). CTACK, also known as CCL27, binds to the CCR10 receptor expressed in normal skin, favoring T-cell homing to the inflammatory microenvironment and thus maintaining immune surveillance. Observational evidence on CTACK and CRC is limited to a single experience demonstrating no statistically significant difference in the expression of CTACK mRNA levels in CRC compared with normal paratumor tissues (68).

Overall, our data on circulating IL6 and CXCL10 expression were consistent with previous observations in CRC patients, whereas we were the first to demonstrate a correlation between plasma levels of either SCGF-β or CTACK and CRC patient clinical outcome.

The correlation between different altered metabolites, highlighted by overlapping and integrated pathways and/or cytokines, might also explain, with the exception of 3-hydroxybutyrate, the lack of association of any single parameter with DFS in the multivariate analysis. Thus, taking advantage of SVM, we built a multiple biomarkers model that, by combining presurgery plasma levels of 3-hydroxybutyrate, cholesterol, phospholipids, triglycerides and IL-6, was able to correctly classify patients by their DFS with good accuracy. Notably, it is important to emphasize that this model appears particularly useful to identify, before surgery, patients with early recurrence and DFS < 1 year who could benefit from a risk-adapted strategy with additional chemotherapy or a shift to an alternative treatment. Similarly, among these patients, more intensive follow-up postsurgery and consolidation by adjuvant treatment should be implemented.

## Conclusions

To the best of our knowledge, our study is the first to perform a combined dynamic evaluation of plasma metabolomics, lipidomics and cytokinomics in metastatic CRC patients undergoing liver resection after induction chemotherapy treatment. The combined analysis of different analytes was able to successfully discriminate presurgical patients at high or low risk of recurrence and provide insight into the associated metabolic and inflammatory processes.

However, our observational and exploratory study has several limitations, including a limited sample size from a single center and a retrospective approach, which might bias the results we have observed. Therefore, these results need to be validated in larger cohorts and prospective studies. The present pilot study indicates the great potential of this combined biomarker approach for defining personalized management strategies in candidate patients for CRCLM resection after induction treatment.

## Data availability statement

The datasets presented in this study can be found in online repositories. The names of the repository/repositories and accession number(s) can be found below: https://gbox.garr.it/garrbox/index.php/s/pIsk5CBjeHgH5DX.

## Ethics statement

The studies involving human participants were reviewed and approved by Comitato Etico Indipendente dell’Istituto Nazionale per lo Studio e la Cura dei Tumori di Napoli - Fondazione Pascale. The patients/participants provided their written informed consent to participate in this study.

## Author contributions

SC, EDG, and FC performed experiments. AB, SC, and AA interpreted the data, carried out statistical analysis and wrote the manuscript. CV, SC, EDG, ADS, GN, SVS, FT, PD, and FI collected patients’ samples and clinical data. AB, AA, and SC designed the study. SC drew the figures. All authors approved the final manuscript. Corresponding authors contributed equally to this work.
