# Supplementary material for: Plasma metabolomics, lipidomics and cytokinomics profiling predict disease recurrence in metastatic colorectal cancer patients undergoing liver resection
Source: Front Oncol. 2023 Jan 11;12:1110104. doi: 10.3389/fonc.2022.1110104 (PMC9875807; doi:10.3389/fonc.2022.1110104)
Supplement: Supplementary file 1 [file DataSheet_1.docx]

Supplementary Material

**Table 1S.** Enriched molecular function analysis on significant metabolites in the plasma metabolome of patients.

| **PathwayName** | **Metabolites** | **p** |
| --- | --- | --- |
| Aminoacyl-tRNA biosynthesis | Histidine, valine, glutamate, isoleucine | 5.54E-01 |
| Butanoate metabolism | 3-hydroxybutyrate, GABA, glutamate | 2.25E+00 |
| Arginine and proline metabolism | Glutamate, GABA, hydroxyproline | 0.0015706 |
| Alanine, aspartate and glutamate metabolism | GABA, glutamate | 0.0025728 |
| Valine, leucine and isoleucine biosynthesis | Valine, isoleucine | 0.0032556 |
| beta-Alanine metabolism | GABA, histidine | 0.0035002 |
| Nitrogenmetabolism | Glutamate, histidine | 0.0067366 |
| Valine, leucine and isoleucine degradation | Valine, isoleucine | 0.0070793 |
| Histidine metabolism | Glutamate, histidine | 0.0085287 |
| Synthesis and degradation of ketone bodies | 3-hydroxybutyrate | 0.019797 |
| D-Glutamine and D-glutamate metabolism | glutamate | 0.036032 |

**Table 2S.** Univariate and Multivariate analyses of baseline patient characteristics, metabolites, lipids and cytokines for overall survival (OS).

|  | **Univariate** | **Multivariate** |
| --- | --- | --- |
|  | **HR (95% CI) P value** | **HR (95% CI) P value** |
| ***Patients characteristics*** |  |  |
| **Gender** (M *vs* F) | 2.53 (0.85-7.57) p=0.053 | - |
| **RAS status** (mutant *vs* wild-type) | 1.67 (0.59-4.76) p=0.37 | - |
| **TRG** (3-4 *vs* 1-2) | 2.18 (0.71-6.70)p=0.12 | - |
| **ARM** (standard *vs* experimental) | 2.42 (0.77-7.53) p=0.38 | - |
| **CEA** (>5 UI/L vs ≤ 5 UI/L) | 1.41 (0.34-5.87) p=0.63 | - |
| **Primary tumor location**(left *vs* right) | 1.33 (0.37-4.82) p=0.46 | - |
| ***Metabolites***(nps) |  |  |
| **3-hydroxybutyrate level** (≥-0.322 *vs*< -0.322) | 5.43 (1.94-15.11) **p=0.002*** | 7.44 (0.83-66.86) **p=0.047*** |
| **histidine level** (<0.158 *vs* ≥0.158) | 4.72 (1.70-13.18) **p=0.023*** | 1.01 (0.28-16.44)p=0.96 |
| ***Lipids***(nps) |  |  |
| **Choline** (<-0.0256 *vs* ≥-0.0256) | 2.82 (0.87-9.23) **p=0.043*** | 1.62 (0.72-2.99)p=0.88 |
|  |  |  |
| **Cholesterol** (≥0.0109 *vs*<0.0109) | 9.08 (3.22-25.59) **p=0.008*** | 1.65 (0.35-3.51)p=0.29 |
| **Triglycerides**(≥-0.000524 *vs*<-0.000524) | 3.31 (1.20-9.15) **p=0.045*** | 2.01 (0.14-29.34)p=0.98 |
| **Phospholipids** (≥-0.147 *vs*<-0.147 | 2.82 (1.01-7.89) **p=0.043*** | 1.03 (0.26-37.72)p=0.58 |
| ***Cytokines***(pg/mL) |  |  |
| **IL-6** (≥5.45 *vs*<5.45**)** | 4.99 (1.74-14.40) **p=0.001*** | 1.34 (0.22-3.01)p=0.99 |
| **SCGF-β **(≥80000*vs*<80000) | 5.61 (1.57-20.06) p=0.06 | - |
| **CXCL10** (≥189 *vs*<189) | 8.63 (2.64-28.24) **p=0.008*** | 2.30 (0.47-21.94) p=0.95 |
| **CTACK** (<6.30 *vs* ≥6.30) | 6.94 (0.62-77.42) **p=0.011*** | 1.76 (0.57-2.96) p=0.45 |

HR: hazard ratio; CI: confidence interval; M: male; F: female; nps: normalized proton signal. Significant p-values <0.05 are reported in bold.

**Table 3S.** Enriched molecular function analysis on significant metabolites in the plasma metabolome of patients at baseline.

| **PathwayName** | **Metabolites** | **p** |
| --- | --- | --- |
| Aminoacyl-tRNA biosynthesis | aspartate, isoleucine ,proline | 0.0020 |
| Arginine and proline metabolism | hydroxyproline, proline | 0.019 |
| Synthesis and degradation of ketone bodies | 3-hydroxybutyrate | 0.029 |
| Valine, leucine and isoleucine biosynthesis | isoleucine | 0.046 |


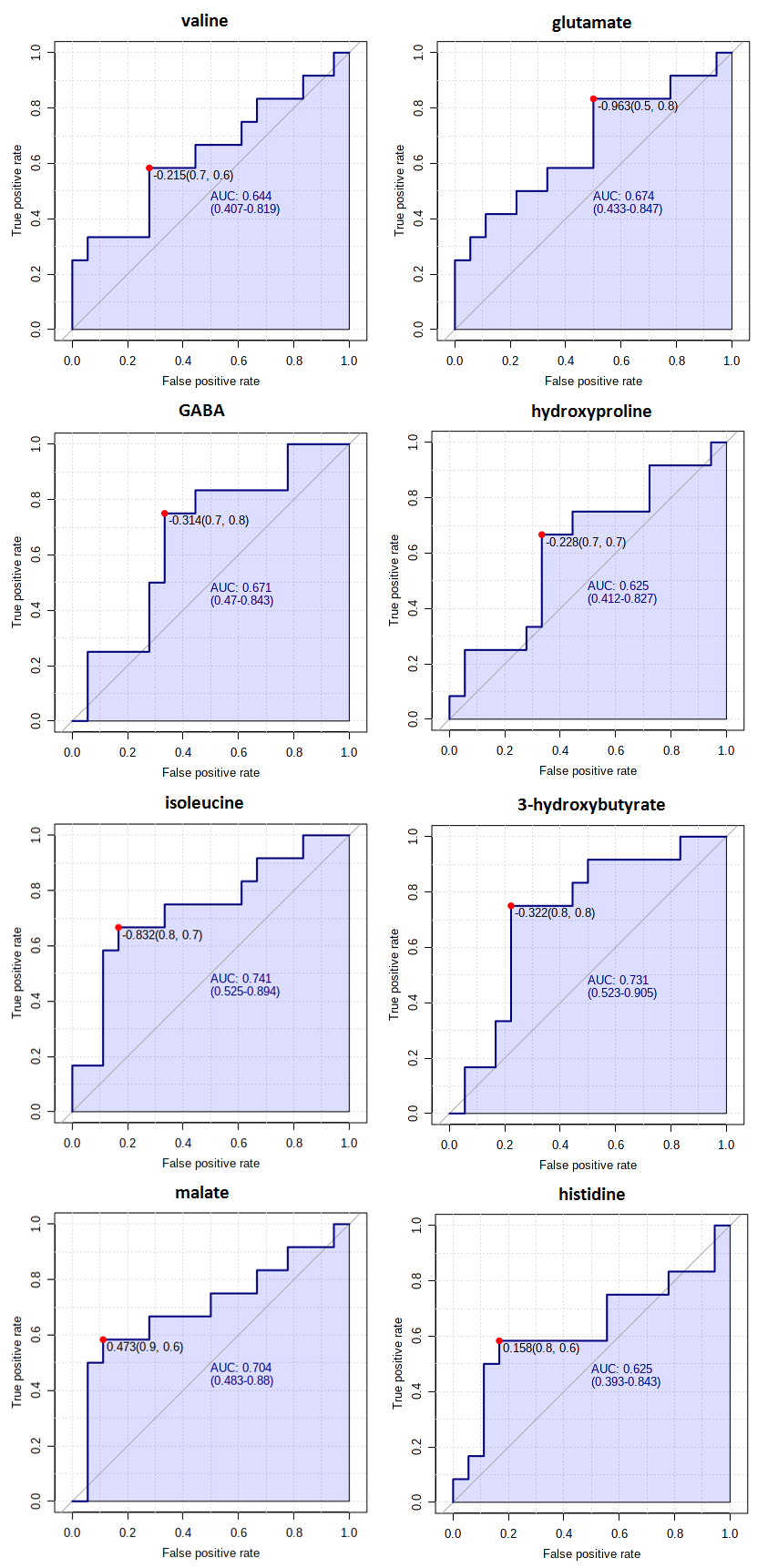


**Figure 1S.**ROC curves performed on the metabolites selected by sPLS-DA. The cutoff values are evidenced by red circles.


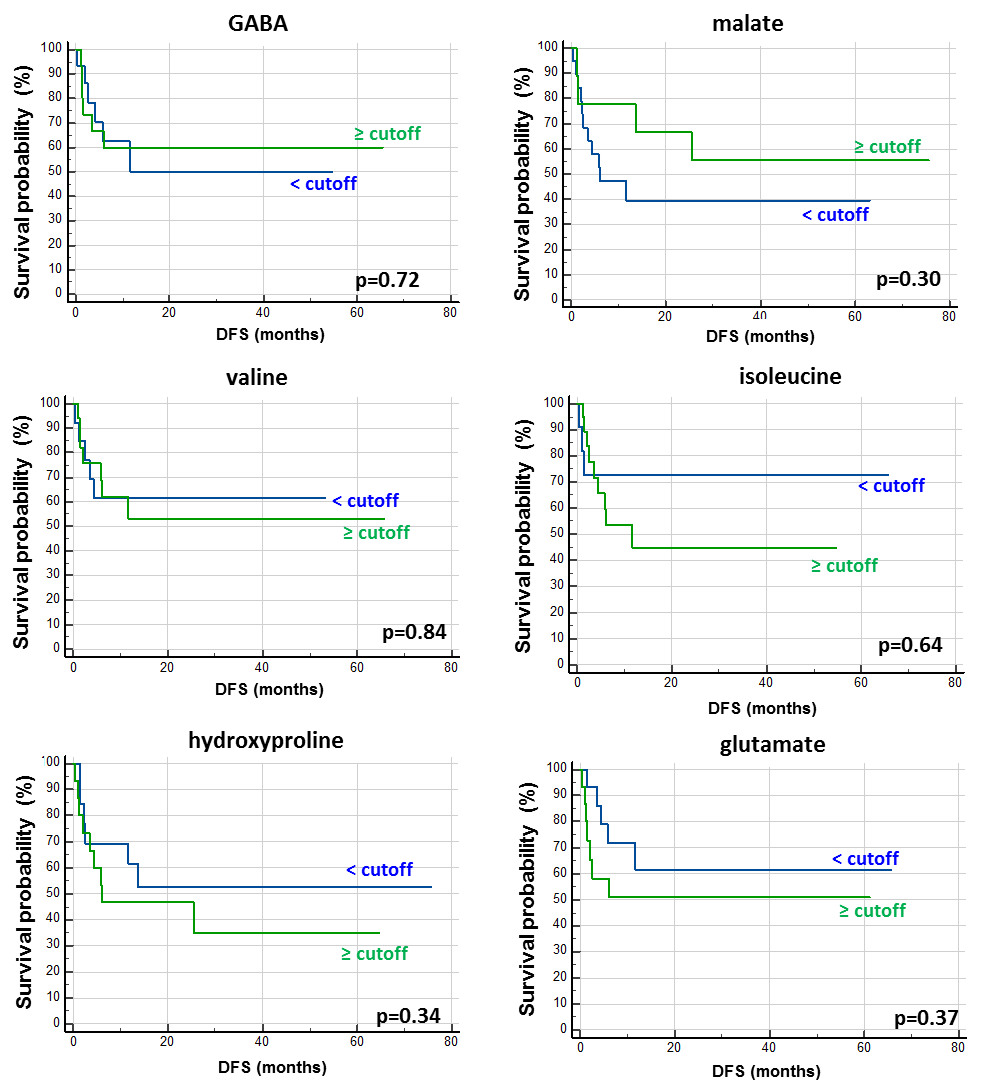


**Figure 2S.** Kaplan–Meier curves of disease free survival (DFS) accordingly to GABA, malate, valine, isoleucine, hydroxyproline and glutamate.

**
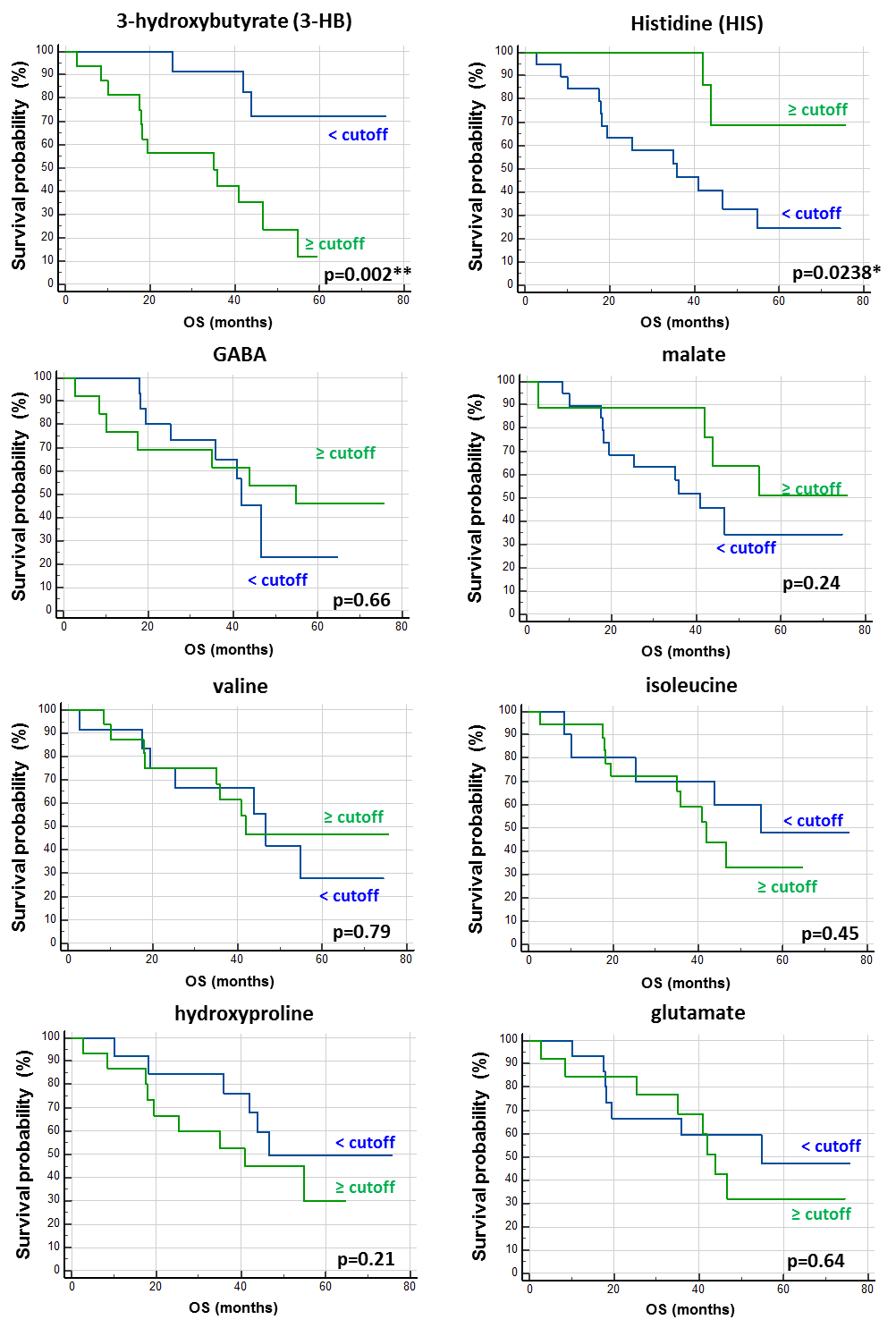
**

**Figure 3S.** Kaplan–Meier curves of overall survival (OS) accordingly to 3-hydroxybutyrate (3-HB), histidine (HIS), GABA, malate, valine, isoleucine, hydroxyproline and glutamate.


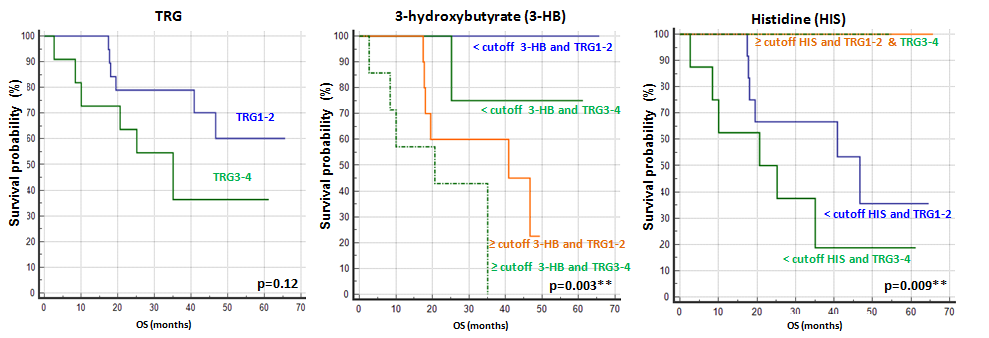


**Figure 4S.** Kaplan–Meier curves of OS accordingly to tumor regression grade (TRG) alone or in combination with either 3-HB or HIS. Log-rank p-values are reported.

**
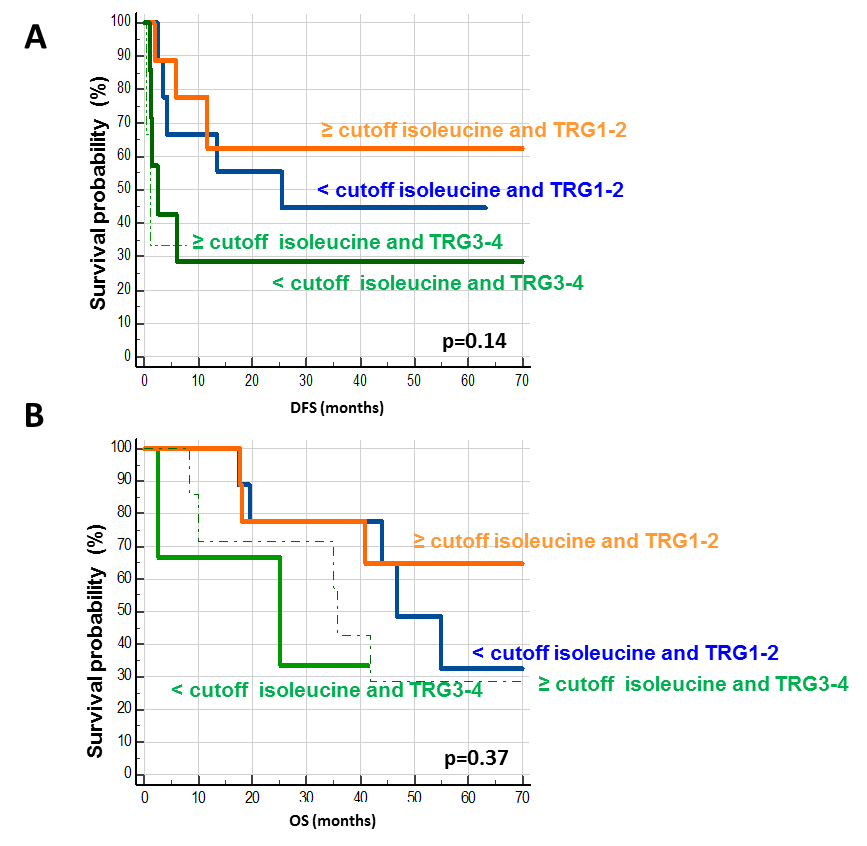
**

**Figure 5S.** Kaplan–Meier curves of DFS (A) and OS (B) accordingly to tumor regression grade (TRG) in combination with isoleucine as control to verify that only histidine and 3-hydroxybutyrate were far better predictors of DFS and OS, with patients with either low 3-hydroxybutyrate or high histidine being associated with more favorable DFS/OS outcomes, independent of TRG status. Log-rank p-value is reported.

**
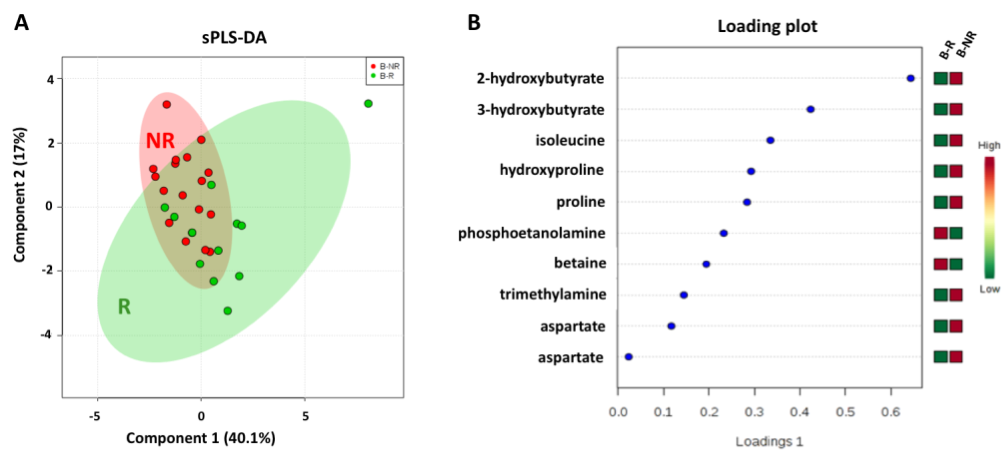
**


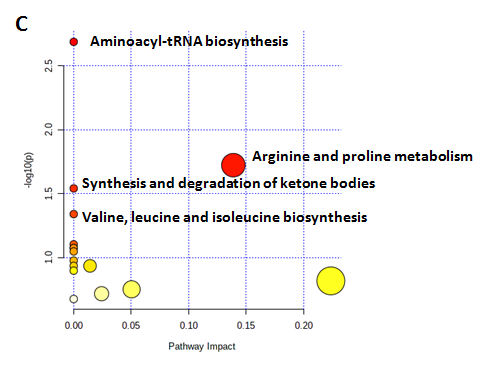


**Figure 6S.** Score plot (A) and loading plot (B) related to metabolomic profiling on plasma of mCRC patients, collected at baseline and subdivided accordingly to DFS in good (B-R; DFS ≥ 1 year) and poor (B-NR; DFS < 1 year) responders. (C) Enriched molecular function analysis on significant metabolites in the plasma metabolome of patients. The most significant pathways are reported: colors, from yellow to red, indicate increasing levels of statistically significance (p values from the pathway enrichment analysis); size of the nodes indicates pathway impact (a combination of both pathway enrichment results and centrality of each of the matched metabolites within the pathway).


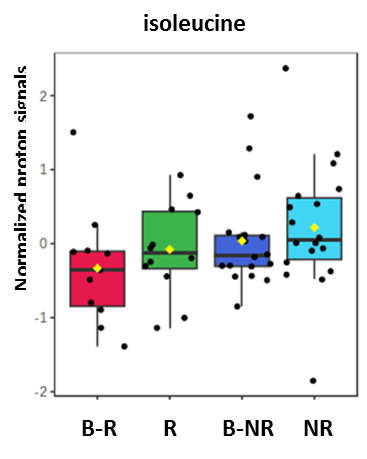


**Figure 7S.** Box and whisker plots summarize the normalized values of isoleucine evaluated at both baseline (B-R and B-NR) and at response evaluation (R and NR).


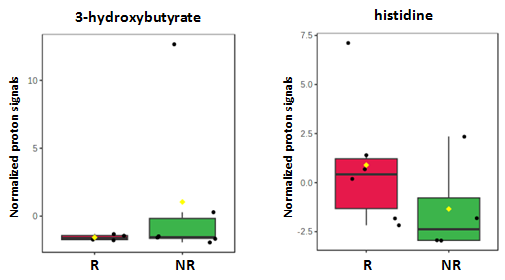


**Figure 8S.** Box and whisker plots summarize the normalized values of 3-hydroxybutyrate and histidine evaluated on resected liver metastases tissues 4 R and 6 NR mCRC subdivided accordingly to DFS in good (R; DFS ≥ 1 year) and bad (NR; DFS < 1 year) responders.


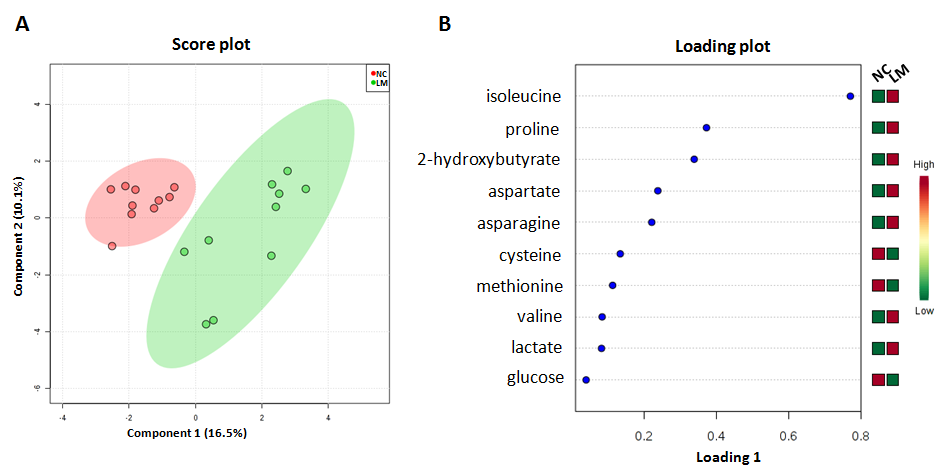
**Figure 9S.** Score plot (**A**) and loading plot (**B**) related to metabolomic profiling, on all 10 (4 R and 6 NR patients) resected liver metastases tissues (LM) and matched non-cancerous adjacent tissues (NC).


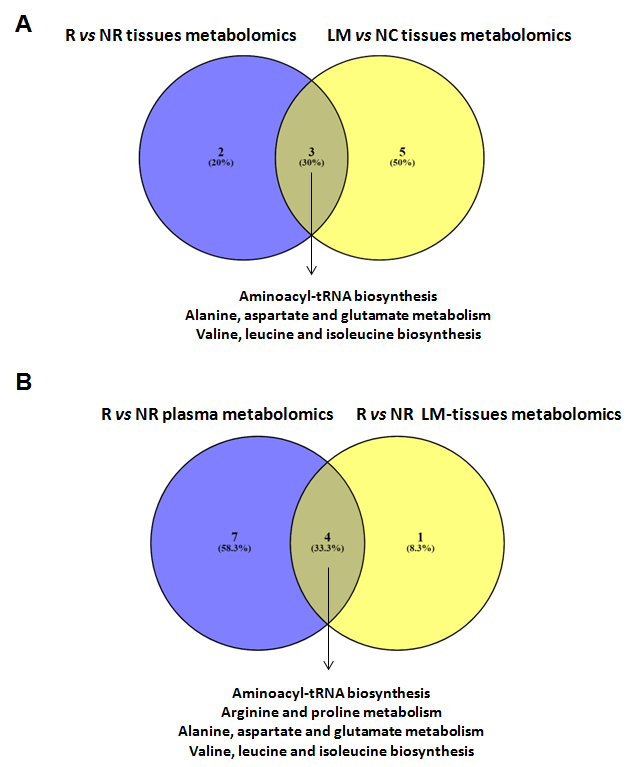


**Figure 10S. A.**Venn diagram reporting metabolite-set enriched molecular analysis based on significant tissue metabolites selected by PLS-DA comparing resected liver metastases (LM) tissues(metabolomics)from R *vs* NR patients and all LM-tissues vs corresponding non-cancerous normal adjacent mucosa (NC). **B**. Venn diagram reporting metabolite-set enriched molecular analysis based on significant plasmaand LM-tissue metabolites selected by PLS-DA comparing R to NR patients.


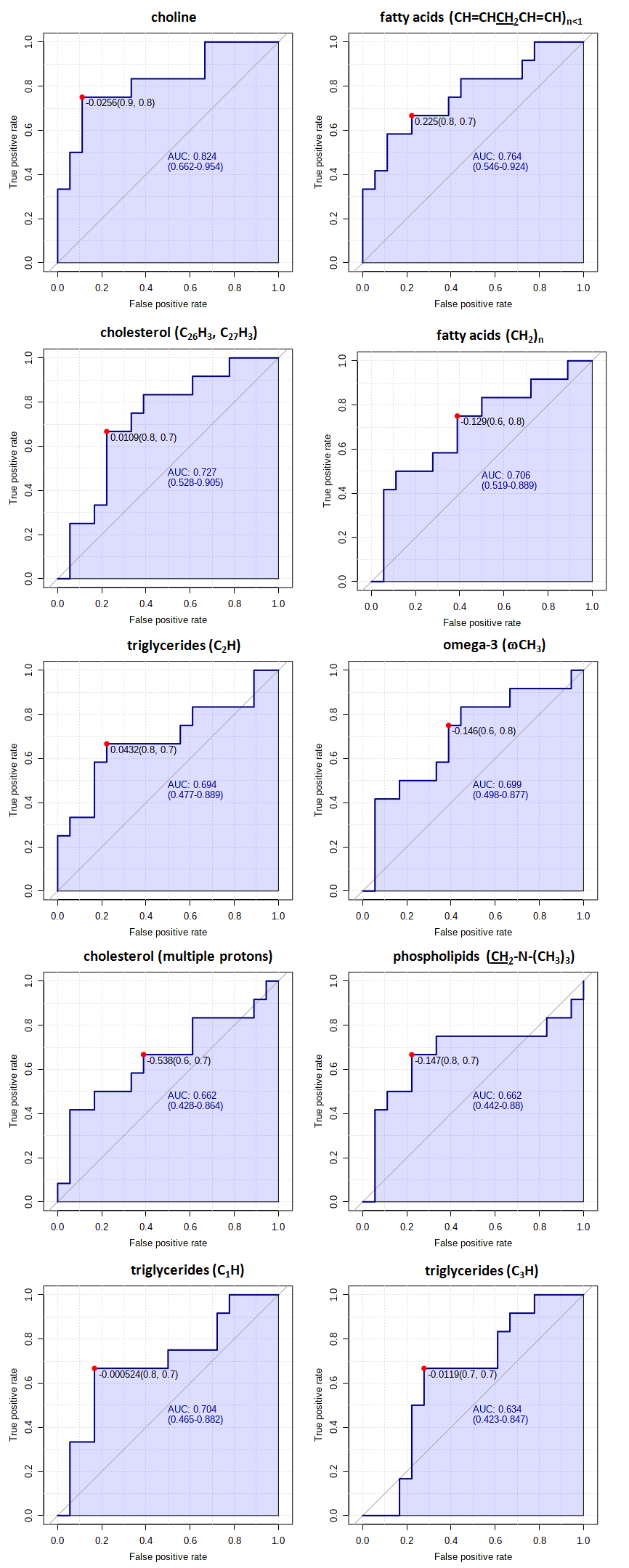


**Figure 11S.** ROC curves performed on the H^1^ NMR lipidic signals selected by sPLS-DA. The cutoff values are evidenced by red circles.


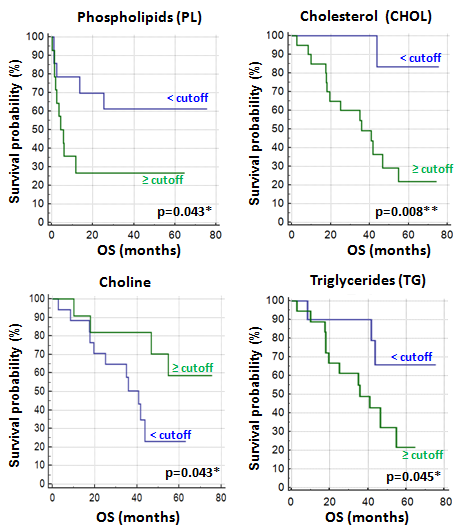


**Figure 12S.** Kaplan–Meier curves of overall survival (OS) accordingly to phospholipids, cholesterol, choline, and triglycerides. Log-rank p-values are reported.


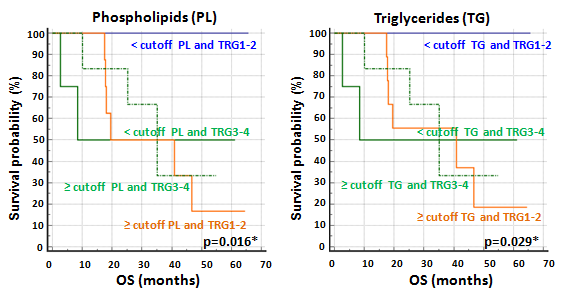


**Figure 13S.** Kaplan–Meier curves of overall survival (OS) accordingly to tumor regression grade (TRG) alone (log-rank p=0.12) and in combination with PL or TG (log-rank p-values are reported).


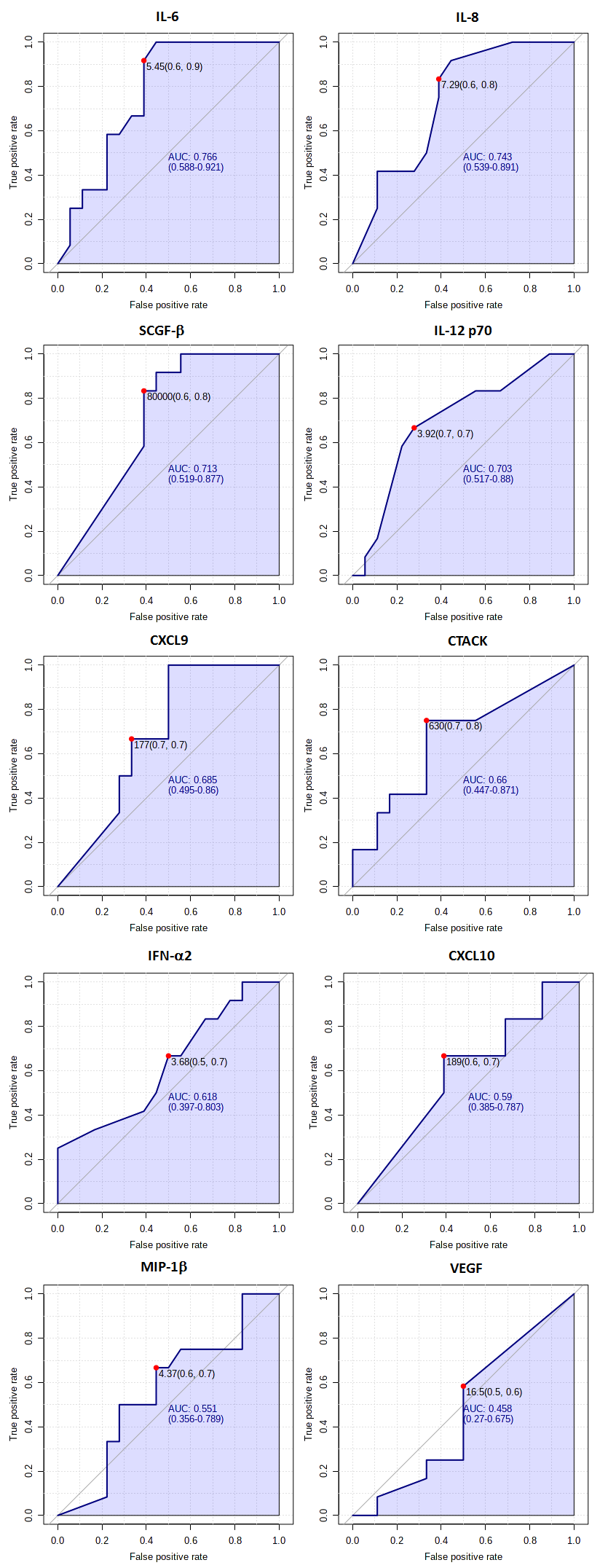
**Figure 14S.** ROC curves evaluated on the significant cytokines in the plasma metabolome of patients subdivided between good (R) and bad (NR) responders. The cutoff values are evidenced by red circles.


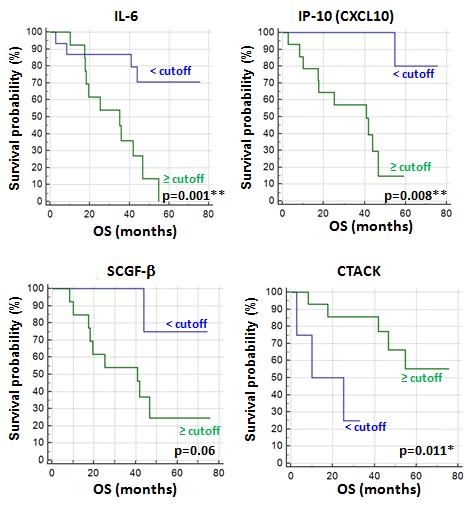


**Figure 15S. A.** Kaplan–Meier curves of overall survival (OS) accordingly to IL-6, CXCL10, SCGF-β and CTACK. Log-rank p-valuesare reported.


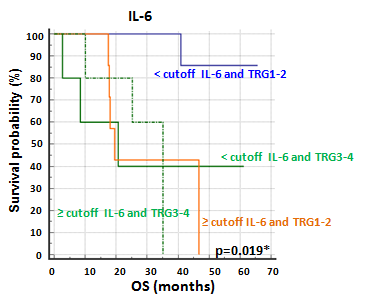


**Figure 16S.** Kaplan–Meier curves of overall survival (OS) accordingly to tumor regression grade (TRG) alone (log-rank p=0.12) and in combination with IL-6 (log-rank p-value is reported).
